# Synthesis and Antiproliferatory Activities Evaluation of Multi-Substituted Isatin Derivatives

**DOI:** 10.3390/molecules26010176

**Published:** 2020-12-31

**Authors:** Ying Ding, Lianbo Zhao, Ying Fu, Lei Hao, Yupeng Fu, Yuan Yuan, Peng Yu, Yuou Teng

**Affiliations:** China International Science and Technology Cooperation Base of Food Nutrition/Safety and Medicinal Chemistry, College of Bioengineering, Tianjin University of Science and Technology, Tianjin 300457, China; dingying9511@163.com (Y.D.); zhaolianbo123456@126.com (L.Z.); fuying@mail.tust.edu.cn (Y.F.); haoleiyes@foxmail.com (L.H.); 15834010536@163.com (Y.F.); yuanyuan@tust.edu.cn (Y.Y.)

**Keywords:** Sandmeyer reaction, multi-substituted isatin derivatives, cytotoxicities, apoptosis, migration, tube formation

## Abstract

A series of multi-substituted isatin derivatives were synthesized using the powerful Sandmeyer reaction. The structures of these derivatives were confirmed by ^1^H-NMR, ^13^C-NMR, and HR-MS. Inhibition of proliferation activities of these derivatives against human leukemia cells (K562), human hepatocellular carcinoma cells (HepG2) and human colon carcinoma cells (HT-29) were evaluated in vitro using the MTT assay. Among the series, compound **4l** exhibited strong antiproliferatory activities against K562, HepG2 and HT-29 cells with IC_50_ values of 1.75, 3.20, and 4.17 μM, respectively. The morphological, growth inhibitory and apoptosic effects of compound **4l** in K562 cells, wound healing effect in HepG2 cells, and tube formating effect in matrix gel of HUVEC cells were evaluated consequently. All results indicated that compound **4l** could be used as a potential antitumor agent in further investigations.

## 1. Introduction

Isatin (ISA) is also known as indole quinone which is widely distributed in mammalian tissues, bodily fluids and plants [1]. Isatin and its derivatives have a variety of biological activities [2] including antitumor activity [3]. For example, isatin is one of the main components of indigo naturalis, a classical and effective antiproliferation Chinese medicine [4]. The anti-tumor activity of isatin has been studied extensively, such as the inhibitory effect of the proliferation of HL60 (human promyelocytic leukemia), N1E-115 (mouse neuroblastoma) and PC12 (rat adrenal pheochromocytoma) cells. The EC_50_ values ranged from 25 to 50 µM [5]. In addition to reducing cell viability, isatin also could induce the DNA fragmentation and chromatin condensation which suggest the apoptosis development [5]. The apoptotic effect of isatin against SH-SY5Y cells was observed at 50 µM in vitro, which exhibited a concentration dependent trend (50, 100 and 200 μM). Further study showed that isatin significantly decreased the level of antiapoptotic protein (Bcl-2) and vascular endothelial growth factor (VEGF) [3,6]. Nagarsenkar [7] found that hybrid Z-8I could trigger the mitochondrial-mediated intrinsic pathway then induce the apoptosis of cancer cells. Further mechanic studies demonstrated that hybrid Z-8I could arrest the G2/M phase of DU145 cell cycle in a dose-dependent manner, leading to an evlevated intracellular reactive oxygen species (ROS) level which may cause the collapse of mitochondrial membrane in DU145 cells. For tumor metastasis, isatin inhibits not only viability of SH-SY5Y cells but also their migration and invasion at 50 µM [8]. Matrix metalloprotein (MMP) are known as an important class of ECM-degrading enzymes, with MMP-2 and MMP-9 correlated with tumor invasive and metastatic [9]. When SH-SY5Y cells were incubated at 100–400 µM, the expression of MMP-2 and MMP-9 decreased. Sunitinib is a multi-target small molecule tyrosine kinase inhibitor developed by Pfizer Pharmaceuticals, it was approved by the FDA in 2006 for treatment of gastrointestinal stromal tumors [10,11]. In addition, SU9516 is a selective inhibitor of cyclin-dependent kinase 2 (CDK2), which can inhibit the proliferation of various tumor cells [12,13]. Therefore, synthesis of small molecule compounds containing isatin structure has been a hot topic in the field of anti-tumor drugs research in recent years.

In medicinal chemistry, the modification of biomolecules with halogens (Cl, Br, I, and F) has been a hot issue. About 40% of halogenated drugs have entered the market or preclinical trial stage, about 25% of organic halogenated drugs have entered the market so far, while 34% of halogenated drugs are still in the development phase [14] which indicates that halogen plays an important role in the drug discovery and development. Among them, fluorine and chlorine are the most widely used, while iodine is relatively rare [15]. Of all the small molecule anti-cancer lead compounds approved by the National Cancer Institute’s Diversity Set IV, 20% of the molecules contain I, Br and Cl [14]. Halogen has the characteristics of increasing molecular lipophilicity, improving permeability to lipid membranes, and electronegativity of halogen can increase central molecule biological activity, etc. [16]. For example, the introduction of strong electron-withdrawing groups such as F can enhance binding, metabolic stability, selectivity, and improve physicochemical properties [17].

In light of the concepts mentioned above, we focused on the synthesis and evaluations of the antiproliferatory activities of multi-substituted isatin derivatives, attempting to promote the research of isatin employment in drug research.

## 2. Results and Discussion

### 2.1. Synthesis of Compounds **4a**-**4o**

**Scheme 1:**
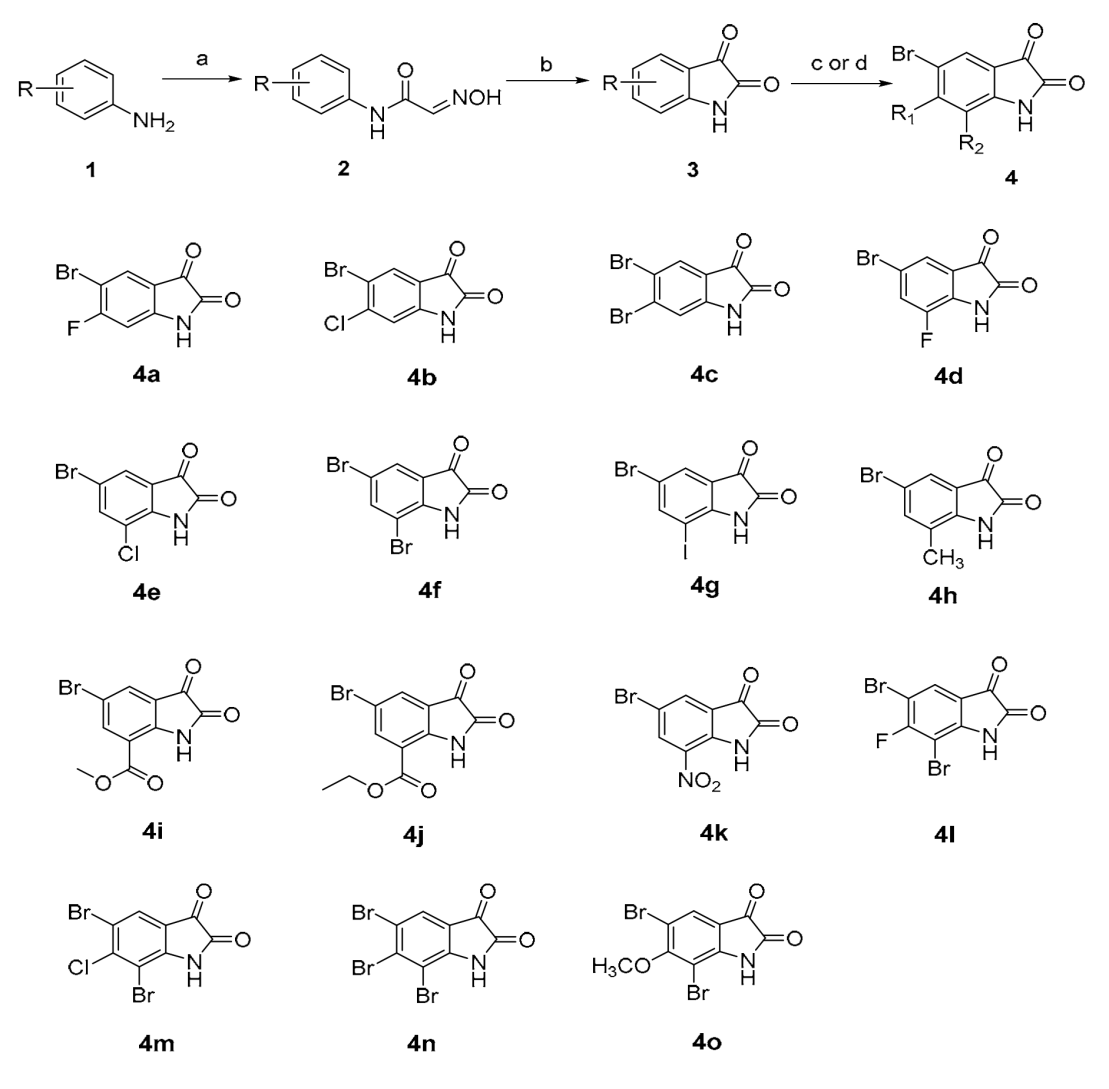
Synthesis of multi-substituted isatin derivatives **4a**–**4o**. Reagents and conditions: (a) Na_2_SO_4_, NH_2_OH HCl, CCl_3_CH(OH)_2_, 2 mol/L HCl, H_2_O, 90 °C, 2 h; (b) conc. H_2_SO_4_, 65 °C, 30 min; (c) Br_2_, AcOH, 90 °C, 2 h; (d) NBS, DMF, 25 °C, 12 h.

A series of multi-substituted isatin derivatives were synthesized using the Sandmeyer reaction as shown in Scheme 1.

The target molecules were synthesized via three steps reaction. In the first step, commercially available aniline derivatives (**1**). Hydroxylamine hydrochloride and chloral hydrate were condensed in acid solution to give intermediate (**2**). Then cyclize by treating with concentrated sulfuric acid to generate isatin derivatives (**3**). In the next step, target compounds **4a**–**4o** were obtained through selective bromination or nitration.

### 2.2. Cytotoxic Effect Against K562 and HepG2 Treated with Compound **4l** In Vitro

In vitro cytotoxicity of compounds **4a**–**4o** were determined against human leukemia cells (K562), human hepatoma cells (HepG2), and human colon cancer cells (HT-29) firstly. The IC_50_ values are summarized in Table 1. The cytotoxic effect of compounds **4a**–**4c** was compared to that of 5-bromoisatin (compound **3f)**, addition of a fluorine atom at the 6-position of **3f** increased the inhibitory activity against leukemia cells (K562), resulting in an IC_50_ value of 2.32 µM. Substitution with bromine or chlorine atom did not increase its activity. Comparing **4d**–**4k** with **3f**, addition of an electron-donating or electron-withdrawing group at the 7-position of **3f** failed to significantly improve their antiproliferative activities. However, bromination at the 7-position (compound **4f**) significantly increased the inhibitory activity against human colon cancer cells (HT-29), with an IC_50_ of 2.67 µM. These results showed that cytotoxic effect on tumor cells was increased by addition of fluoride at the 6-position or bromide at the 7-position of **3f**. Next, compounds **4l**–**4o**, a series of tri-substituted isatin derivatives, were synthesized. The results showed substitution at the 5-, 6- and 7-positions with halogens resulted in higher inhibitory activity against leukemia cells (K562). Among these compounds, compound **4l** exhibited the highest inhibitory activity against all three cell types, with IC_50_ values of 1.75, 3.20, and 4.17 µM. Based on these results, we conducted further mechanistic studies of compound **4l**. 

### 2.3. Cell Proliferation Assay of Compound **4l**

To determine the sensitivity of cell lines on compound **4l**, we tested the growth inhibition activity of compound **4l** in vitro using MTT method. Human leukemia cells (K562), human liver cancer cells (HepG2), human colon cancer cells (HT-29; HCT-116), breast cancer cells (MDA-MB-231), prostate cancer cells (PC-3), human lung cancer cells (A549), normal human renal epithelial cells (293T), and normal human umbilical vein endothelial cells (HUVEC) were selected for cell proliferation assay. As shown in Table 2, compound **4l** exerted excellent inhibitory effects against K562 (IC_50_ = 1.75 μM), HepG2 (IC_50_ = 3.20 μM) cells and HT-29 (IC_50_ = 4.17 μM) cells in vitro. The IC_50_ values for compound **4l** against 293T cells were greater than the maximum test concentration (100 μM) and the IC_50_ value for HUVEC cells was 61.83 μM, which indicated that the toxicity of compound **4l** against normal cell lines was much less than that against cancer cell lines.

### 2.4. Morphological Changes of K562 Cells Treated with Compound **4l**

**Figure 1:**
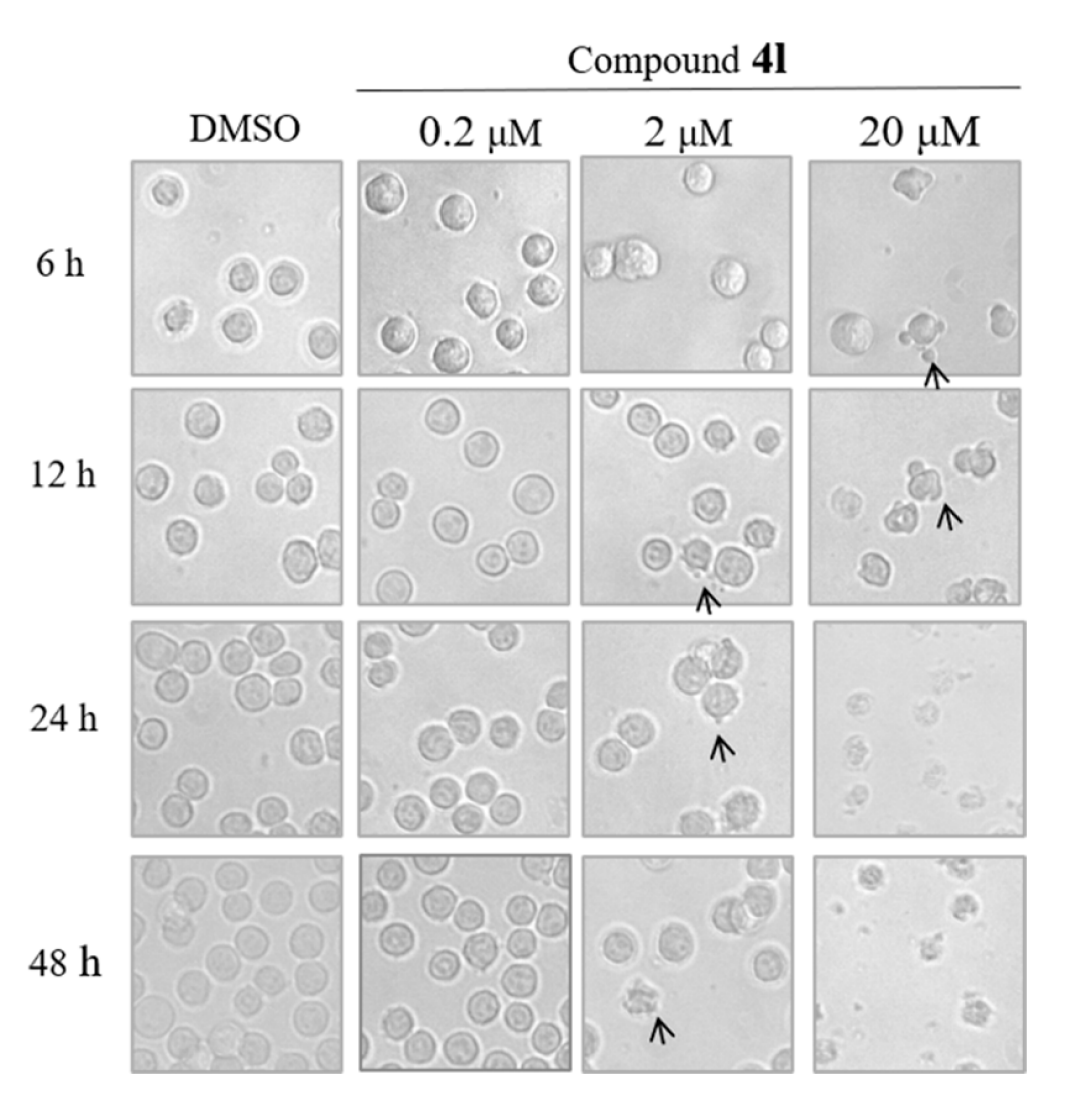
Morphological observation changes in compound **4l** treated K562 cells by different concentration (0, 0.2, 2, 20 μM) and different period of time (6, 12, 24, 48 h).

Two main pathways of cell death are necrosis and apoptosis. Raising apoptosis rate is considered one way for cancer treatment [18]. The adjacent interstitial cells or macrophages of this complex are rapidly engulfed, and no inflammation occurs in the surrounding tissues. Therefore, light microscopy was used to observe the in vitro morphological changes of cultured cells, such as cell shrinkage and formation of apoptotic bodies [19]. As the arrows point in Figure 1, compared with the positive control DMSO (substitute for 0 μM), 0.2 μM compound **4l** did not induce apoptosis bodies. However, apoptosis bodies were observed at 2 μM, and more extensive apoptosis bodies occurred at 20 μM, which demonstrated concentration dependence. K562 cells treated with compound **4l** (6 h, 20 μM) had increased apoptotic bodies at 12 h. The cell surface shrank at 24 h and 48 h in response to 2 μM compound **4l**. These results indicated that after treatment of compound **4l** cell volume gradually decreases during the early stage of apoptosis, the chromatin in the nucleus condenses, apoptotic bodies appear on the surface of K562 cells.

### 2.5. Compound **4l** Inhibited K562 Cell Apoptosis

**Figure 2:**
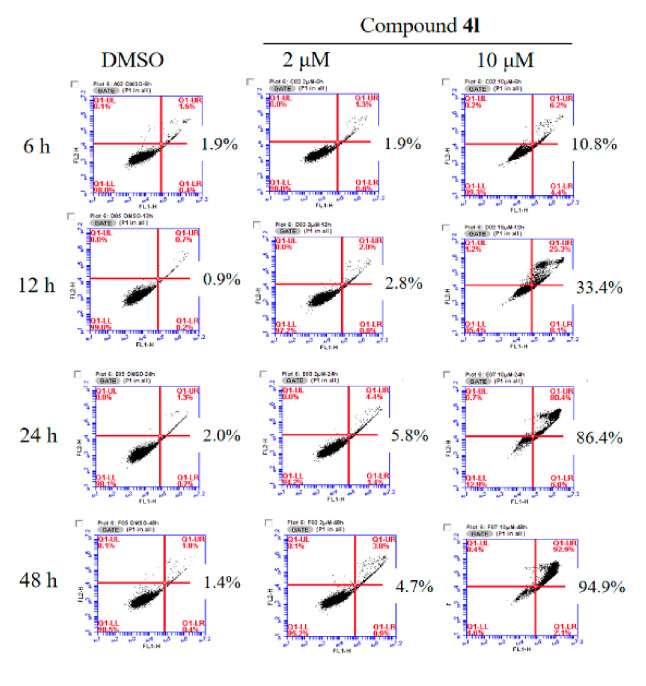
Apoptosis observation changes in compound **4l** treated K562 cells by different concentration (0, 2, 10 μM) and by different period of time (6, 12, 24, 48 h).

Through the previous study, compound **4l** greatly influenced the morphology of K562 cells, resulting in cell shrinkage, cell membrane sprouting, irregular round shape, and formation of apoptotic bodies of different sizes. To further investigate whether compound **4l** inhibits tumor cell proliferation through cell apoptosis pathway, Annexin-V-FITC and PI staining assay was performed on K562 cells [20]. As shown in the Figure 2, compound **4l** increased apoptosis in K562 cells compared with DMSO treatment in a time-dependent manner. Treatment with 10 μM compound **4l** increased the percentage of apoptotic cells to 10.8%, 33.4%, 86.4%, and 94.9% at 6, 12, 24 and 48 h, respectively. Thus, we can conclude that antitumor activity of compound **4l** was achieved through inducing apoptosis.

### 2.6. Compound **4l** Inhibited K562 Cell Proliferation

**Figure 3:**
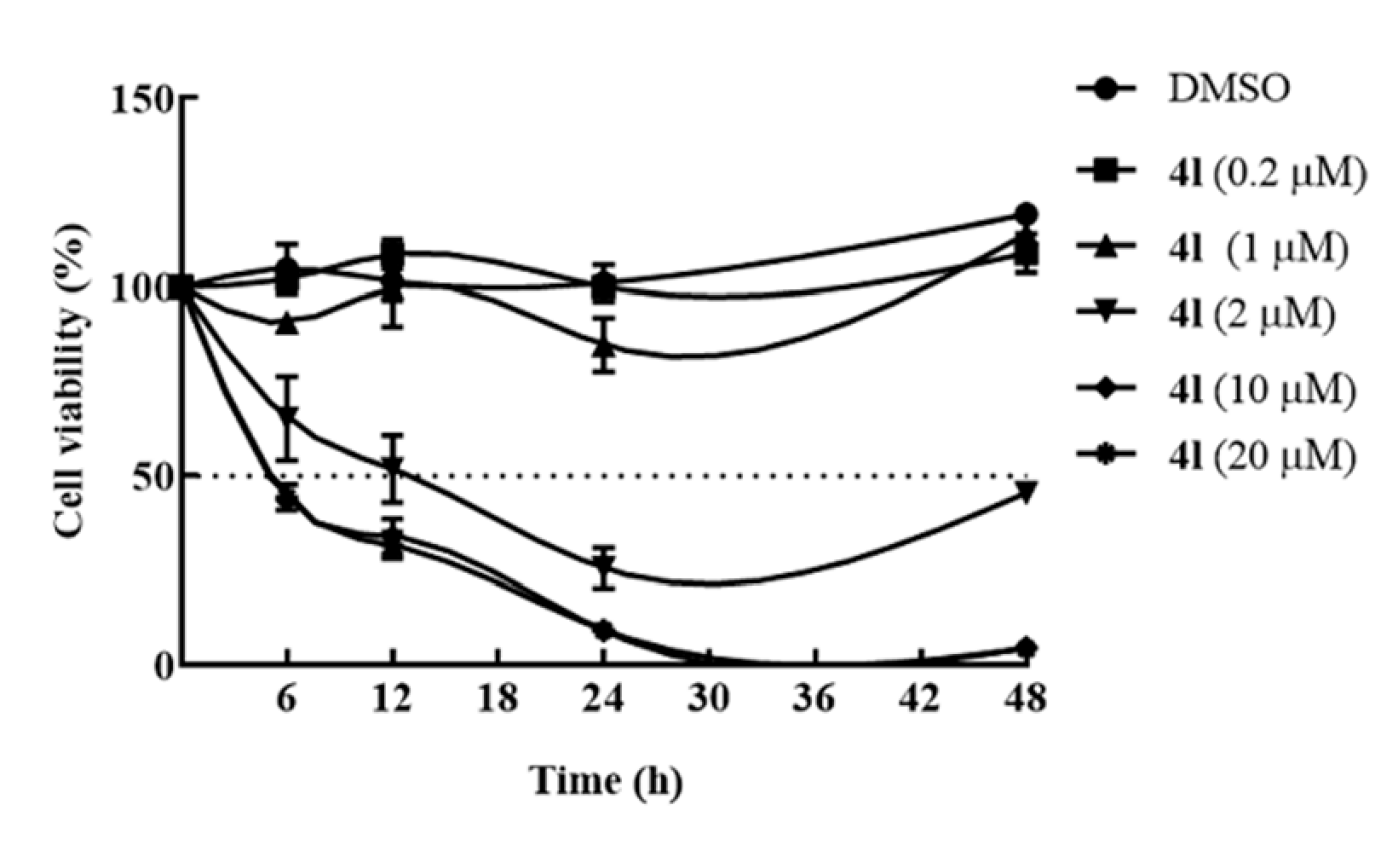
The growth curve of compound **4l** on K562 cells at 6, 12, 24, 28 h, cell viability was defined as 100% at 0 h.

In order to determine the relationship between the time and concentration of compound **4l** action on K562 cells, the growth curve was further drawn. Compound **4l** inhibited proliferation of K562 cells. The time course of compound **4l** inhibition of K562 cells for 6, 12, 24 and 48 h at 0.2, 1, 2, 10, and 20 μM was evaluated. As shown in Figure 3, the inhibitory effect of compound 4l on proliferation of K562 cells was time-and concentration-dependent at 2, 10 and 20 μM. However, incubation with 0.2 and 1 μM compound **4l** did not result in further growth inhibition.

### 2.7. Compound **4l** Suppressed Cell Migration in HepG2 Cells and Tube Formation in HUVECs

**Figure 4:**
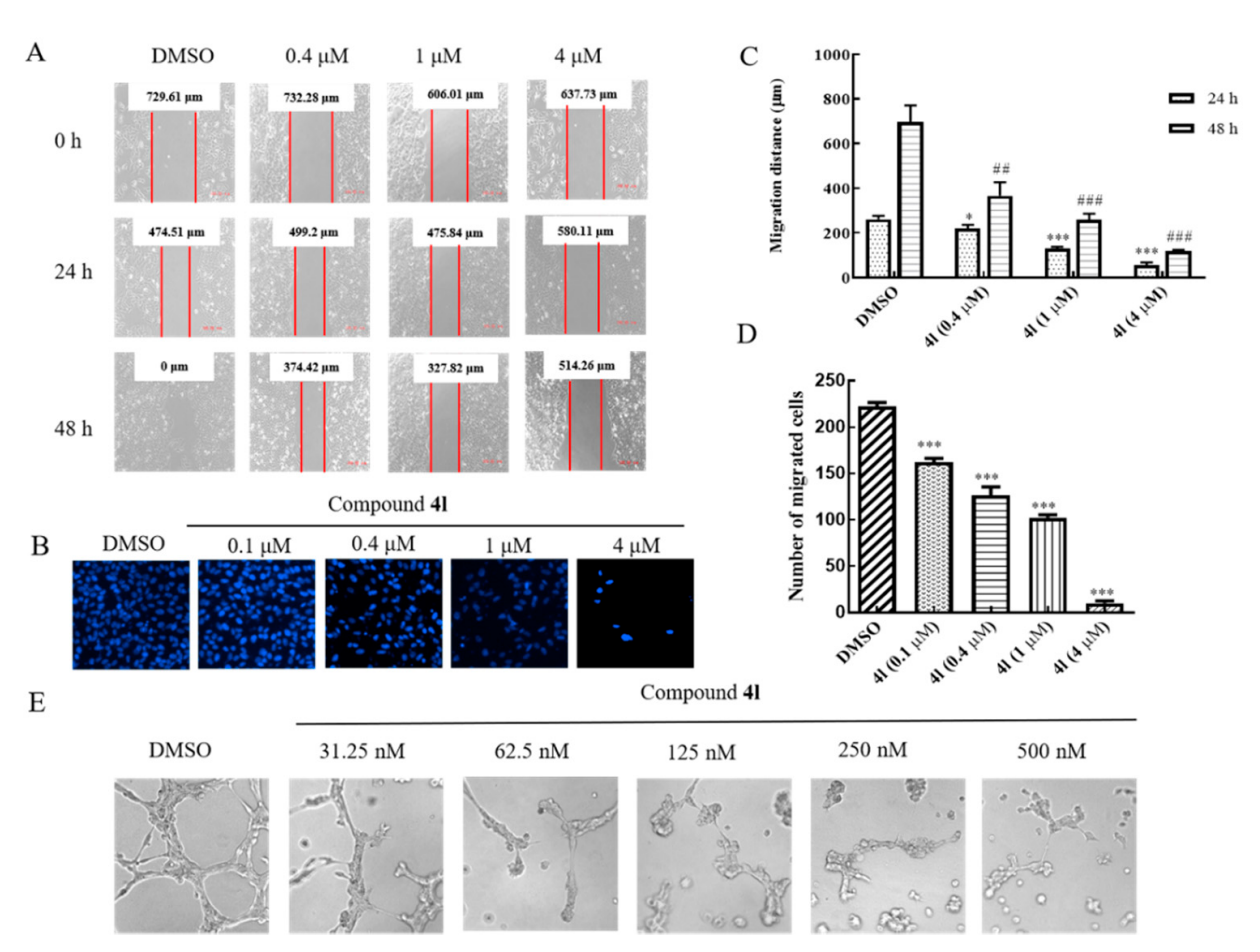
Effect of compound **4l** on HepG2 cells migration and on HUVECs tube formation. (**A**) Using 0, 0.4, 1, 4 μM contration for wound healing assay. (**B**) Using 0, 0.1, 0.4, 1 and 4 μM concentration for transwell assay, taking 100-fold photographs under an inverted microscope. (**C**) Transverse migration distance. (**D**) Number of migrated cells at different concentrations. (**E**) Detection of the ability of compound **4l** to tube formation on matrix glue. Compound concentrations ranging from 31.25 to 500 nM inhibited tube formation of HUVEC cells. (* *p* < 0.5 versus DMSO, *** *p* < 0.001 versus DMSO, ^##^ *p* < 0.01 versus DMSO, ^###^ *p* < 0.001 versus DMSO, Compound **4l** test.).

Tumor cells cultured in vitro are invasive in that they exhibit the expansive behavior of tumor cells, which are characterized by migratory and invasive behavior. Migration of cancer cells also reflects metastatic ability, and physiologically significant [21]. In order to study the inhibitory effect of compound **4l** on the migration ability of HepG2 cells, the cell scratch repair and transwell methods were used. The results showed that the wound healing rate was very fast in the DMSO control group, but compound **4l** inhibited migration and growth of HepG2 cells was retarded. The results in Figure 4A,C showed that increasing concentrations of compound **4l** (0.4, 1 and 4 μM), resulted in increased inhibition of migration, with the most significant inhibition in response to 4 μM compound **4l**. After 48 h the cells in DMSO group had completely covered the scratched region.

The transwell method was used to study the effect of compound **4l** on longitudinal migration of HepG2 cells. Compound **4l** was added to HepG2 cells (1 × 10^5^ cells/mL) at different concentrations 12 h after plating. The results in Figure 4B,D showed that compound **4l** significantly inhibited longitudinal migration of HepG2 cells after 24 h in a concentration-dependent manner. Thus, our results indicate that compound **4l** utilized here can inhibit migration of HepG2 cells.

### 2.8. Compound **4l** Suppressed Tube Formation in HUVECs

Tube forming treatment of tumor is a strategy aimed at tumor vascular system. Angiogenesis in adult individuals occurs under pathological conditions, such as during inflammation and the immune response, tumorigenesis, metastasis, and injury repair [22]. As shown in the Figure 4E, HUVEC cells were inoculated on matrix gel at a density of 2 × 10^5^ cells/mL. Dense reticular structure was formed on the matrix gel in the DMSO group (31.25, 62.5, 125 and 500 nM). Compound **4l** at 31.25 nM significantly inhibited tube formation *in HUVECs* after 12 h. The results further revealed that the compound **4l** not only inhibited the proliferation of tumor cells, but also on tumor angiogenesis.

## 3. Conclusions

In our study, a series of novel multi-substituted isatin derivatives were designed and synthesized, the antiproliferatory activity was evaluated then, compound **4l** was screened out with excellent inhibitory activity against K562, HepG2 and HT-29 cells with IC_50_ values of 1.75, 3.20, and 4.17 μM, also with lower toxicity toward normal cells (293T) than CPT. The anti-tumor mechanism of compound **4l** was preliminarily studied in vitro, using morphology observation, growth curve drawing and apoptosis assay of K562 cells, migration assay of HepG2 cells and angiogenesis assay of HUVEC cells to prove that compound **4l** can inhibit the growth of tumor cell, cause tumor cell apoptosis, prevent migration and tube formation. The results support that compound **4l** could be used as a potential antitumor candidate in future investigations.

## 4. Materials and Methods

### 4.1. General

All commercial materials and reagents are not further purified for use unless otherwise specified. All the solvents used are pre-treated by distillation. The solvents for reaction were distilled to remove water over Na or CaH_2_. All reactions are required to carry out in the dry, noble gas (nitrogen or argon) protected atmosphere. Column chromatography was employed as the purification method, silica mesh number being of 200-300, Qingdao, China. The ^1^H and ^13^C NMR spectra were recorded at 400 MHz and 100 MHz with a Bruker AM-400 MHz NMR spectrometer (Billerica, Middlesex, MA, USA). The chemical shift of the hydrogen spectrum of the compounds (ppm) were given according to different deuterium agents(CDCl_3_ δ = 7.26 ppm, DMSO-d_6_ δ = 2.50 ppm quoted proton signal). Hertz (Hz) represents the coupling constant. NMR spectra were recorded on a Brüker AM-400 MHz using CDCl_3_ or CD_3_SOCD_3_ signal as internal reference (CDCl_3_: δ (^1^H) =7.26 ppm and δ (^13^C) = 77.16 ppm, CD_3_SOCD_3_: δ (^1^H) = 2.50 ppm and δ (^13^C) = 39.51 ppm). In NMR peak signals, the following abbreviations are denoted as s = singlet, d = dualet, t = triplet, m = multiple, and dd = doublet of doublets. ESI mass spectra were obtained on an LCQ-Advantayc MAX (LAM10188, Thermo Finnigan, Co., Ltd., Pleasanton, CA, USA). The reported products were confirmed by comparing their corresponding ^1^H NMR and ^13^C NMR spectra with counterparts reported in the literatures.

#### 4.1.1. General Procedure I: Synthesis of Compound **3**

A mixture of substituted aniline (compound **1**) (0.045 mol), hydroxylamine hydrochloride (0.15 mol), Na_2_SO_4_ (0.35 mol) and 5 mL hydrochloric acid (2 mol/L) were stirred in 250 mL H_2_O. The resulting mixture was stirred at 25 °C for 5 min, and additional chloral hydrate (0.05 mol) was added, then the mixture was heated to reflux at 90 °C for 2 h. After the reaction finished, the reaction produced a mixture, which was filtered and dried. The crude product was used directly for the next step without further purification.

The *N*-2-(hydroxyimino) acetamide derivatives (compound **2**, 0.04 mol) was added to a flask (100 mL) which contained concentrated sulfuric acid (15 mL) at 50 °C with vigorous stirring. After the addition, the mixture was heated to 80 °C and stirred for 30 min. The mixture was poured into ice-water mixture, and the resultant precipitate was filtered, the precipitate was dried in vacuo to yield the crude which was purified by dissolving in dilute sodium hydroxide (5%, 100 mL) followed by acidified with 4N hydrochloric acid (20 mL). The solid which formed was filtered out and dried over air to provide the purified compounds **3 [23]**.

#### 4.1.2. General Procedure II: Synthesis of Multi-Substituted Isatin Derivatives (**4a**–**4c**)

To a solution of compound **3** (3.0 mmol) in *N*, *N*-dimethylformamide (2.5 mL) was added NBS (3.3 mmol). The resulting reaction mixture was stirred at 25 °C for 12 h. After the reaction finished, the mixture was diluted with water (10 mL), the resultant precipitate filtered, and the precipitate was dried in vacuo. The crude product was chromatographed gradiently on silica gel with PE/EA (10:1−4:1) to give the compounds **4a**–**4c**.

#### 4.1.3. General Procedure III: Synthesis of Multi-Substituted Isatin Derivatives (**4d**–**4j**)

To a cold (0 °C) suspension of compound **3** (3.0 mmol) in acetic acid (5 mL) was added Br_2_ (6.0 mmol) dropwise. After the addition, the reaction was warmed to reflux until the reaction is complete as indicated by TLC. The reaction solution was then poured into water (10 mL), and the precipitate was filtered, dried in vacuo. The crude product was chromatographed gradiently on silica gel with PE/EA (10:1−4:1) to give the compounds **4d**–**4j**.

#### 4.1.4. General Procedure IV: Synthesis of Multi-Substituted Isatin Derivatives (**4l**–**4o**)

To a cold (0 °C) suspension of compound **3** (1.1 mmol) in acetic acid (10 mL) was added Br_2_ (4.4 mmol) dropwise. After the addition, the reaction was warmed to reflux until the reaction is complete as indicated by TLC. The reaction solution was then poured into water (10 mL), and the precipitate was filtered, dried in vacuo. The crude product was chromatographed gradiently on silica gel with PE/EA (20:1−5:1) to give the compounds **4l**–**4o**(see ^1^H-NMR, ^13^C-NMR spectra of compounds **4l**–**4o** in Supplementary Materials).

**5-bromo-6-fluoro isatin (4a)**. Following General Procedure II, using compound **3a** (500 mg, 3.00 mmol) and NBS (590 mg, 3.30 mmol), compound **4a** was obtained (550 mg, 74% yield) as a red solid; ^1^H NMR (400 MHz, DMSO-*d*_6_) *δ* 11.29 (s, 1H), 7.89 (d, *J* = 7.2 Hz, 1H), 6.93 (d, *J* = 9.2 Hz, 1H). ^13^C NMR (100 MHz, DMSO-*d*_6_) *δ* 182.0, 164.0 (d, *J* = 253 Hz), 159.7, 152.4 (d, *J* = 13 Hz), 130.3 (d, *J* = 3 Hz), 116.4 (d, *J* = 3 Hz), 102.1 (d, *J* = 28 Hz), 101.7 (d, *J* = 23 Hz); HR-MS-ESI (m/z) *calcd. for C8H3NO2FBr* [M+H]^+^, 244.0173; found 244.0161.

**5-bromo-6-chloro isatin (4b)**. Following General Procedure II, using compound **3b** (1.0 g, 5.50 mmol) and NBS (1.08 g, 6.10 mmol), compound **4b** was obtained (1.0 g, 71% yield) as a red solid; ^1^H NMR (400 MHz, DMSO-*d*_6_) *δ* 11.27 (s, 1H), 7.88 (s, 1H), 7.13 (s, 1H). ^13^C NMR (100 MHz, DMSO-*d*_6_) *δ* 182.6, 159.5, 150.7, 142.0, 129.5, 118.8, 114.9, 114.4; HR-MS-ESI (m/z) *calcd. for C8H3BrClNO2* [M+H]^+^, 260.4719; found 260.4705.

**5, 6-dibromo isatin (4c)**. Ref. [24] Following General Procedure II, using compound **3c** (500 mg, 2.20 mmol) and NBS (430 mg, 2.40 mmol), compound **4c** was obtained (440 mg, 76% yield) as a red solid; ^1^H NMR (400 MHz, DMSO-*d*_6_) *δ* 11.23 (s, 1H), 7.84 (s, 1H), 7.26 (s, 1H). ^13^C NMR (100 MHz, DMSO-*d*_6_) *δ* 182.8, 159.4, 150.3, 133.7, 129.1, 119.2, 117.5, 117.3; HR-MS-ESI (m/z) *calcd. for C8H3Br2NO2* [M+H]^+^, 304.9229; found 304.9213.

**5-bromo-7-fluoro isatin (4d)**. Following General Procedure III, using compound **3d** (500 mg, 3.0 mmol) and Br_2_ (970 mg, 6.0 mmol), compound **4d** was obtained (380 mg, 51% yield) as a red solid; ^1^H NMR (400 MHz, DMSO-*d*_6_) *δ* 11.67 (s, 1H), 7.86-7.89 (m, 1H), 7.56 (s, 1H). ^13^C NMR (100 MHz, DMSO-*d*_6_) *δ* 182.3 (d, *J* = 4 Hz), 159.3, 147.7 (d, *J* = 249 Hz), 137.3 (d, *J* = 14 Hz), 127.4 (d, *J* = 21 Hz), 123.6 (d, *J* = 4 Hz), 122.1 (d, *J* = 5 Hz), 114.0 (d, *J* = 7 Hz); HR-MS-ESI (m/z) *calcd. for C8H3NO2FBr* [M+H]^+^, 244.0173; found 244.0158.

**5-bromo-7-chloro isatin (4e)**. Ref. [25] Following General Procedure III, using compound **3e** (1.0 g, 5.50 mmol) and Br_2_ (1.80 g, 6.0 mmol), compound **4e** was obtained (570 mg, 79% yield) as a red solid; ^1^H NMR (400 MHz, DMSO-*d*_6_) *δ* 11.59 (s, 1H), 7.93 (s, 1H), 7.65 (s, 1H). ^13^C NMR (100 MHz, DMSO-*d*_6_) *δ* 182.6, 159.7, 147.4, 138.9, 126.0, 121.5, 117.8, 114.7; HR-MS-ESI (m/z) *calcd. for C8H3NO2ClBr* [M+H]^+^, 260.4719; found 260.4703.

**5, 7-dibromo isatin (4f)**. Ref. [24] Following General Procedure III, using compound **3f** (1.0 g, 4.40 mmol) and Br_2_ (1.40 g, 8.80 mmol), compound **4f** was obtained (1.16 g, 86% yield) as a red solid; ^1^H NMR (400 MHz, DMSO-*d*_6_) *δ* 11.44 (s, 1H), 8.05 (s, 1H), 7.68 (s, 1H). ^13^C NMR (100 MHz, DMSO-*d*_6_) *δ* 182.9, 159.9, 149.0, 141.6, 126.4, 121.7, 115.1, 106.2; HR-MS-ESI (m/z) *calcd. for C8H3NO2Br2* [M+H]^+^, 304.9229; found 304.9212.

**5-bromo-7-iodo isatin (4g).** Following General Procedure III, using compound **3g** (1.0 g, 3.70 mmol) and Br_2_ (1.20 g, 7.40 mmol), compound **4g** was obtained (520 mg, 40% yield) as a red solid; ^1^H NMR (400 MHz, DMSO-*d*_6_) *δ* 11.13 (s, 1H), 8.14 (s, 1H), 7.66 (s, 1H). ^13^C NMR (100 MHz, DMSO-*d*_6_) *δ* 183.8, 160.1, 152.6, 147.3, 126.7, 121.2, 115.5, 80.4; HR-MS-ESI (m/z) *calcd. for C8H3NO2FBr* [M+H]^+^, 351.9187; found 351.9168.

**5-bromo-7-methyl isatin (4h).** Following General Procedure III, using compound **3h** (1.0 g, 6.20 mmol) and Br_2_ (2.0 g, 12.40 mmol), compound **4h** was obtained (750 mg, 50% yield) as a red solid; ^1^H NMR (400 MHz, DMSO-*d*_6_) *δ* 11.22 (s, 1H), 7.65 (s, 1H), 7.35 (s, 1H), 2.27 (s, 3H). ^13^C NMR (100 MHz, DMSO-*d*_6_) *δ* 184.0, 160.0, 148.9, 141.2, 124.8, 124.6, 119.5, 114.7, 15.64; HR-MS-ESI (m/z) *calcd. for C8H3NO2FBr* [M+H]^+^, 240.0625; found 240.0606.

**5-bromo-7-methyl formate isatin (4i).** Following General Procedure III, using compound **3i** (500 mg, 2.40 mmol) and Br_2_ (780 mg, 4.80 mmol), compound **4i** was obtained (180 mg, 26% yield) as a red solid; ^1^H NMR (400 MHz, DMSO-*d*_6_) *δ* 11.03 (s, 1H), 8.09 (s, 1H), 7.93 (s, 1H), 3.90 (s, 3H). ^13^C NMR (100 MHz, DMSO-*d*_6_) *δ* 182.2, 164.0, 159.8, 149.4, 139.3, 131.5, 122.1, 115.5, 114.3, 53.1; HR-MS-ESI (m/z) *calcd. for C10H6NO4Br* [M+H]^+^, 282.9534; found 282.9523.

**5-bromo-7-ethyl formate isatin (4j).** Following General Procedure III, using compound **3j** (500 mg, 2.30 mmol) and Br_2_ (730 mg, 4.60 mmol), compound **4j** was obtained (230 mg, 34% yield) as a yellow solid; ^1^H NMR (400 MHz, DMSO-*d*_6_) *δ* 11.95 (s, 1H), 8.08 (s, 1H), 7.92 (s, 1H), 4.36-4.14 (m, 2H), 1.33 (t, *J* = 6.8 Hz, 3H). ^13^C NMR (100 MHz, DMSO-*d*_6_) *δ* 182.1, 163.5, 159.7, 149.6, 139.2, 131.4, 122.0, 115.6, 114.2, 62.1, 14.6; HR-MS-ESI (m/z) *calcd. for C11H8NO4Br* [M+H]^+^, 296.9634; found 296.9622.

**5-bromo-7-nitro isatin (4k)**. Ref. [26] To a cold (0 °C) suspension of **3f** (1.0 g, 4.40 mmol) in sulfuric acid (10 mL) was added a solution of potassium nitrate (0.45 g, 0.9 mol/L in sulfuric acid) dropwise. After the addition, the reaction was warmed to 25 °C until the reaction is complete as indicated by TLC. The reaction mixture was diluted with ethyl acetate and water. The separated water phase was extracted with ethyl acetate three times, and the combined organic phase was washed with brine, dried over Na_2_SO_4_, concentrated in vacuo, and chromatographed gradiently on silica gel with PE/EA (3:1) to give compound **4k** (610 mg, 51% yield) as a yellow solid; ^1^H NMR (400 MHz, DMSO-*d*_6_) *δ* 11.82 (s, 1H), 8.43 (s,1H), 8.11 (s,1H). ^13^C NMR (100 MHz, DMSO-*d*_6_) *δ* 180.7, 160.0, 144.5, 132.1, 133.0, 132.9, 123.7, 113.7; HR-MS-ESI (m/z) *calcd. for C8H3N2O3Br* [M+H]^+^, 271.0243; found 271.0233.

**5, 7-dibromo-6-fluoro isatin (4l).** Following General Procedure IV, using compound **3a** (500 mg, 3.0 mmol) and Br_2_ (1.94 g, 12.10 mmol), compound **4l** was obtained (820 mg, 84% yield) as a red solid; ^1^H NMR (400 MHz, DMSO-*d*_6_) *δ* 11.66 (s, 1H), 7.91 (d, *J* = 6.8 Hz, 1H). ^13^C NMR (100 MHz, DMSO-*d*_6_) *δ* 181.7, 160.4 (d, *J* = 249 Hz), 160.1, 151.7 (d, *J* = 5 Hz), 128.8 (d, *J* = 2 Hz), 117.4 (d, *J* = 3 Hz), 101.9 (d, *J* = 24 Hz), 94.5 (d, *J* = 28 Hz); HR-MS-ESI (m/z) *calcd. for C8H3NO2FBr2* [M+H]^+^, 319.8364; found 319.8339.

**5, 7-dibromo-6-chloro isatin (4m).** Following General Procedure IV, using compound **3b** (1.0 g, 5.50 mmol) and Br_2_ (3.52 g, 22.20 mmol), compound **4m** was obtained (1.42 g, 76% yield) as a red solid; ^1^H NMR (400 MHz, DMSO-*d*_6_) *δ* 11.56 (s, 1H), 7.90 (s, 1H). ^13^C NMR (100 MHz, DMSO-*d*_6_) δ 182.4, 160.1, 150.5, 141.5, 127.9, 119.7, 115.5, 107.1; HR-MS-ESI (m/z) *calcd. for C8H3NO2ClBr* [M+H]^+^, 335.8068; found 335.8049.

**5, 6, 7-tribromo isatin (4n).** Ref. [26] Following General Procedure IV, using compound 3c (1.0 g, 4.40 mmol) and Br2 (2.83 g, 17.70 mmol), compound 4n was obtained (1.22 g, 72% yield) as a red solid; 1H NMR (400 MHz, DMSO-d6) δ 11.50 (s, 1H), 7.85 (s, 1H). 13C NMR (100 MHz, DMSO-d6) δ 182.6, 160.1, 149.9, 136.1, 127.4, 120.2, 118.1, 109.54; HR-MS-ESI (m/z) *calcd. for C8H3NO2Br3* [M+H]^+^, 383.8190; found 383.8175.

**5, 7-dibromo-6-methoxy isatin (4o).** Following General Procedure IV, using compound **3k** (200 mg, 1.10 mmol) and Br_2_ (0.72 g, 4.50 mmol), compound **4o** was obtained (210 mg, 56% yield) as a red solid; ^1^H NMR (400 MHz, DMSO-*d*_6_) *δ* 11.46 (s, 1H), 7.81 (s, 1H), 3.88 (s, 3H). ^13^C NMR (100 MHz, DMSO-*d*_6_) *δ* 182.2, 160.8, 160.3, 151.5, 128.6, 117.4, 110.4, 102.2, 61.3; [M+H]^+^. HR-MS-ESI (m/z) *calcd. for C9H5NO3Br2* [M+H]^+^, 331.8563; found 331.8547.

### 4.2. Biology

#### 4.2.1. Cell Lines and Culture Conditions

The human leukemia K562 cell line were cultureded in RPMI-1640 which purchased from the Shanghai Institutes of Biological Sciences (Shanghai, China).The umbilical vein endothelial cells (HUVECs) were purchased from ATCC (American type culture collection, USA) and cultured in Ham’s F-12 medium.The liver cancer cells (HepG2) were grown in Dulbecco’s modified Eagle’s medium. All cell lines supplemented with 10% fetal bovine serum, 2.05 mM glutamine and 1% penicillin/streptomycin which both were incubated in a humidified atmosphere of 5% CO_2_ at 37 °C. 

#### 4.2.2. MTT Assay for Cytotoxicity

K562 cells, HepG2, HT-29, HCT-116, MDA-MB-231, PC-3, A549, 293T and HUVEC cells, 5 × 10^4^ cells/mL with 10 % fetal bovine serum cell culture medium, 100 μL cell suspension into each hole of 96-well plate, cultured K562 cells for 2 h, HepG2, HT-29, HCT-116, MDA-MB-231, PC-3, A549, 293T and HUVEC cells for 24 h, in 37 °C incubator. Dissolve compound 4l in DMSO, 0.01, 0.1, 1, 10, 100 μM, 0.5 μL/hole, after 48 h, 20 μL MTT (5 mg/mL) was added to each hole. Isopropyl hydrochloride was added to K562 cells, HepG2, HT-29, HCT-116, MDA-MB-231, PC-3, A549, 293T and HUVEC cells were added with DMSO to dissolve the cells. 570 and 630 nm of K562 cell, 490 and 630 nm of other cell lines for OD value which measured by enzyme labeling instrument, than the IC_50_ value is calculated according to OD value.

#### 4.2.3. Apoptosis Analysis

Phosphatidylserine (PS) on cell membrane was labeled with fluorescent dye (Annexin V-FITC & PI) to detect apoptosis. Inoculated K562 cells into 6-well plate, 5 × 10^4^ cells/well, 2 mL per hole. In order to collect enough cells in later stage, multiple holes could be set up. (24 h in 37 °C, 5% CO_2_ incubator). Final 0 μM (replaced by 0.5% DMSO), 0.2 μM and 2 μM compound 4l were added respectively. After 6, 12, 24 and 48 h, centrifuged at 1000 rpm/5 min, K562 cells were evenly dispersed by 1 × PBS which pre-cooled at 4 °C, centrifuged repeat twice as before, supernatant of the culture medium was quickly poured out. The process needs to be operated on ice, adding 100 μL 1× Binding Buffer to suspended cells in centrifugal tube, transferring cell suspension to 1.5 mL EP tube, adding 5 μL Annexin V-FITC, gently shaking and mixing, incubating at 25 °C for 5 min; then adding 5 μL PI solution (20 ug/mL), shaking and mixing, 400 μL 1× Binding Buffer before measuring by flow cytometry. Test by flow cytometric immediately, the experiment should be completed within 1 h. This method can distinguish early and late apoptotic cells which is one of the convenient and reliable methods for quantitative detection of apoptotic cells.

#### 4.2.4. Cell Growth Curve Experiment

K562 cells were inoculated on 96-well plate (5 × 10^4^ cells/mL, 100 μL). Cell suspension was added into each hole, control and blank holes were set at the same time (37 °C and 5% CO_2_ incubator for 24h). Added compound 4l (0.2, 1, 2, 10 and 20 μM) 0.5 μL for each hole, three compound holes were set for each drug concentration. Set both blank group and control group treated with complete medium and DMSO at the same concentration separately. After 6 h, 12 h, 24 h and 48 h of incubation in 37 °C and 5% CO_2_ incubator, MTT 20 μL of 5 mg/mL was added to each pore and incubated in 37 °C and 5% CO_2_ incubator for 4 h, the culture was terminated. Wiping off invalid supernatant carefully, add 100 μL isopropanol hydrochloride into each hole, blow and mix repeatedly. After 10 min at 37 °C, the purple crystalline armor was fully dissolved. The OD value of each pore was measured by enzyme labeling instrument. The following formula is applied for calculating the cell survival rate.

(1) $$Cell\;viability\left(\%\right)=\frac{Drug\;group\;OD-Blank\;group\;OD}{Solvent\;control\;group\;OD-Blank\;group\;OD}\times 100\%$$

#### 4.2.5. Wound Healing Assay

HepG2 cells were inoculated in 12-well dishes (1 × 10^5^ cells/mL, 37 °C, 5% CO_2_ incubators). After adherence, remove the middle part of the cells to create scratches, the cells were removed from the compartment. Dissolve compound **4l** (0.4, 1 and 4 μM) in 1 mL medium. The cells were observed and photographed at different time (0, 24, 48 h), and the width of the middle area was counted. 

#### 4.2.6. Transwell Assay

Firstly, placed transwell chamber in a 24-well plate, then HepG2 cell was injected into the upper chamber with serum-free medium (1 × 10^5^ cells/mL, 200 μL), simultaneously 600 μL 10% fetal bovine serum was based on the lower chamber. When cells adhered on the bottom of the dish, 0.5% DMSO as control, 0.1, 0.4, 1, 4 μM compound **4l** were added to test group, (37 °C, 5% CO_2_, 24 h in incubator) suck out the culture media in both upper and lower chambers. Used 1×PBS to washed both side of the chamber then fixed cells passing through the upper compartment with pure methanol (25 °C, 30 min). Methanol was then sucked out and used 1 × PBS to remove unstable cells. 1 ug/mL DAPI was added to the cell chamber and orifice plate, then placed in the incubator to stain the cells for 15 min. Upper layer non-migrating cells were gently wiped off with a wet cotton swab, excess stain was washed with 1 × PBS to infiltrate the cells. Finally, we observed and photographed staining of nucleus under fluorescence microscope, and counted five visual fields randomly.

#### 4.2.7. Tube Forming Assay

The frozen matri-gel (13.9 mg mL; BD Bioscience, San Jose, CA, USA) is removed from the refrigerator at −20 °C in advance, sealed on ice overnight at 4 °C to dissolve into liquid state. On the second day, 96-well pre-cooled plates were taken out and 50 μL matrix collagen solution was added to each hole, which was shaken gently to distribute evenly in all parts of the hole. Bubbles should be avoided during the process. Then the culture plate was incubated in a incubator at 37 °C for 30 min to stabilize and solidify the matrix collagen solution. Digested HUVEC cells with 0.25% trypsinase and blown evenly to form a single cell suspension, 2 × 10^5^ cells/mL was inoculated. After cell adherence, the drug-adding group added compound **4l** (0 nM, replaced by 0.5% DMSO, 31.25, 62.5, 125, 500 nM), respectively, (5% CO_2_, 12 h in the incubator). Finally, the arrangement and integrity of the tubular structure of cells in each group were observed under inverted microscope then photographed by a Nikon Eclipse Ti microscope (Nikon, Tokyo, Japan).

#### 4.2.8. Statistical Analysis

The data was expressed as the mean ± SD for graphical representation. Analyzed the migration data statistically using Graphpad Prism 7. One-way analysis of *t*-test was used to analyze the data for significance. The analysis was done by comparing the treated groups with untreated control group (DMSO group). The significant results showing * *p* < 0.5 versus DMSO, *** p < 0.001 versus DMSO, ^##^ *p* < 0.01 versus DMSO, ^###^ *p* < 0.001 versus DMSO, compound **4l** test.

## Figures and Tables

**Table 1:** In vitro cytotoxic effect of isatin derivatives.

Compound	IC_50_ (µM) ^a^		
	K562	HepG2	HT-29
**CPT** ^b^	0.03 ± 0.01	0.04 ± 0.01	0.06 ± 0.01
**3f**	>10	>10	>10
**4a**	2.32 ± 0.22	22.93 ± 2.18	15.26 ± 0.24
**4b**	15.16 ± 1.63	28.22 ± 2.06	31.93 ± 1.37
**4c**	12.75 ± 1.38	26.07 ± 2.15	2.14 ± 0.15
**4d**	30.22 ± 0.52	>100	>100
**4e**	11.14 ± 1.23	14.45 ± 1.36	30.83 ± 0.80
**4f**	21.36 ± 2.58	23.52 ± 2.01	2.67 ± 0.20
**4g**	23.38 ± 1.57	30.68 ± 2.39	31.78 ± 1.21
**4h**	20.27 ± 2.02	>100	>100
**4i**	34.61 ± 0.92	33.74 ± 1.06	26.11 ± 0.32
**4j**	73.53 ± 4.16	>100	93.2 ± 2.01
**4k**	>100	>100	>100
**4l**	1.75 ± 0.16	3.20 ± 0.14	4.17 ± 0.33
**4m**	5.05 ± 0.48	36.93 ± 1.26	37.26 ± 0.60
**4n**	2.85 ± 0.95	33.34 ± 1.92	3.68 ± 0.32
**4o**	34.24 ± 0.58	52.70 ± 3.20	42.84 ± 2.98

^a^ IC_50_ values are the mean ± SD of three separate experiments, ^b^ positive control.

**Table 2:** In vitro cell proliferation inhibitory activities of compound **4l** and **CPT**.

Cell Line	IC_50_ (μM)	
	Compound 4l	CPT
Human leukemia (K562)	1.75 ± 0.16	0.03 ± 0.01
Liver cancer (HepG2)	3.20 ± 0.14	0.04 ± 0.01
Human Colon cancer (HT-29)	4.17 ± 0.33	0.06 ± 0.01
Colon cancer (HCT-116)	6.18 ± 0.22	0.24 ± 0.07
Cancer cells (MDA-MB-231)	>100	>100
Human Prostate cancer (PC-3)	>100	>100
Lung cancer (A549)	18.94 ± 1.03	>100
Renal epithelial cells (293T)	>100	9.84 ± 1.81
Umbilical vein endothelial cells (HUVEC)	61.83 ± 2.24	>100

## Data Availability

The data presented in this study are available on request from the corresponding author.

## Supplementary Materials

The following are available online. ^1^H-NMR, ^13^C-NMR of the synthesized compounds **4a**-**4o**, and HR-MS spectra of the synthesized compounds **4l**, **4m** and **4o**.

## Sample Availability

Samples of the compounds are not available from the authors.
