# Supplementary material for: Synthesis and Antiproliferatory Activities Evaluation of Multi-Substituted Isatin Derivatives
Source: Molecules. 2020 Dec 31;26(1):176. doi: 10.3390/molecules26010176 (PMC7795683; doi:10.3390/molecules26010176)
Supplement: Supplementary file 1 [file molecules-26-00176-s001.pdf]

**Supporting information**

# Synthesis and Antiproliferation Activities Evaluation of Multi-substituted Isatin Derivatives

Ying Ding[a, †], Lianbo Zhao [a, †], Ying Fu[a], Lei Hao[a], Yupeng Fu[a], Yuan Yuan[a], Peng Yu[a,*], Yuou Teng[a,*]

[a.] China International Science and Technology Cooperation Base of Food Nutrition/Safety and Medicinal Chemistry, Tianjin University of Science and Technology, Tianjin 300457.

† These authors contributed equally to this work.

* Corresponding author.

## Supplementary content:

1. Structure of the Intermediates……………………..……..…..………..………S2
2. Spetrum Data……………………..……...…..…………………………….……S3-17
3. HR-MS Spectrum Data of new compound **4l,4m,4o**……………..…S18-S20

# 1. Structure of the Intermediates

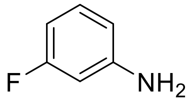
1a

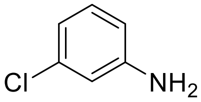
1b

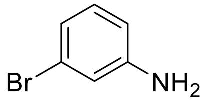
1c

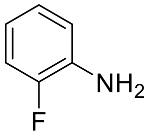
1d

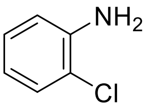
1e

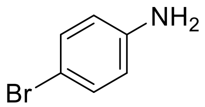
1f

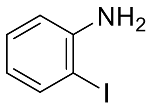
1g

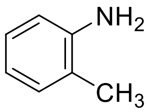
1h

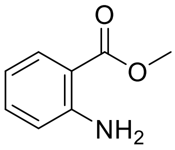
1i

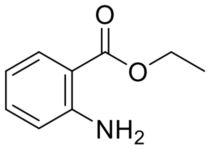
1j

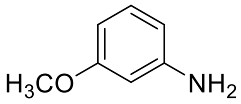
1k

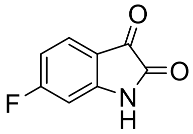
**3a**

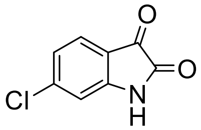
**3b**

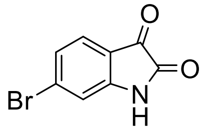
**3c**

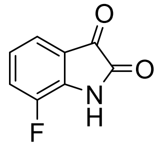
**3d**

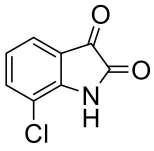
**3e**

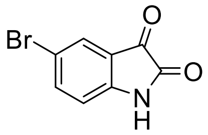
**3f**

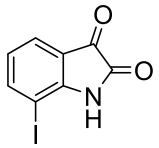
**3g**

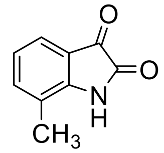
**3h**

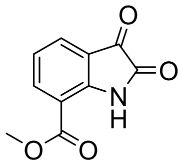
**3i**

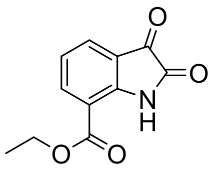
**3j**

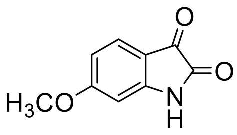
**3k**

# 2. Spectra Data

Spectra data are shown from the next page.

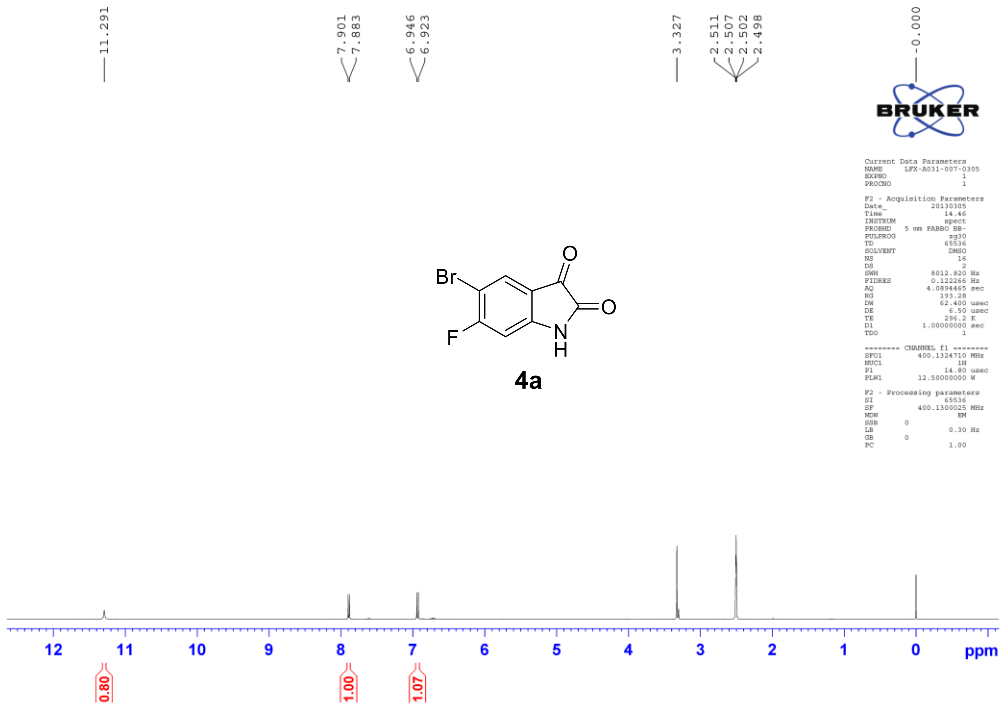
11.291
7.901
7.883
6.946
6.923
3.327
2.511
2.507
2.502
2.498
-0.000
4a
12 11 10 9 8 7 6 5 4 3 2 1 0 ppm
0.80
1.00
1.07

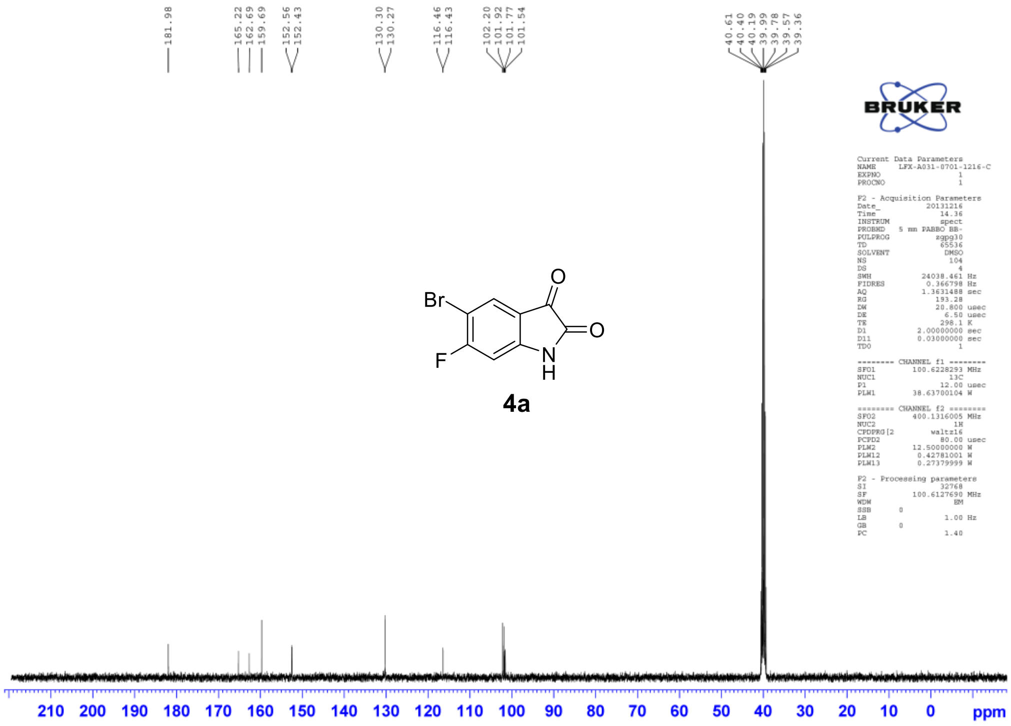
181.98
165.22
162.69
159.69
152.56
152.43
130.30
130.27
116.46
116.43
102.20
101.92
101.77
101.54
40.61
40.40
40.19
39.99
39.78
39.57
39.36
4a
210 200 190 180 170 160 150 140 130 120 110 100 90 80 70 60 50 40 30 20 10 0 ppm

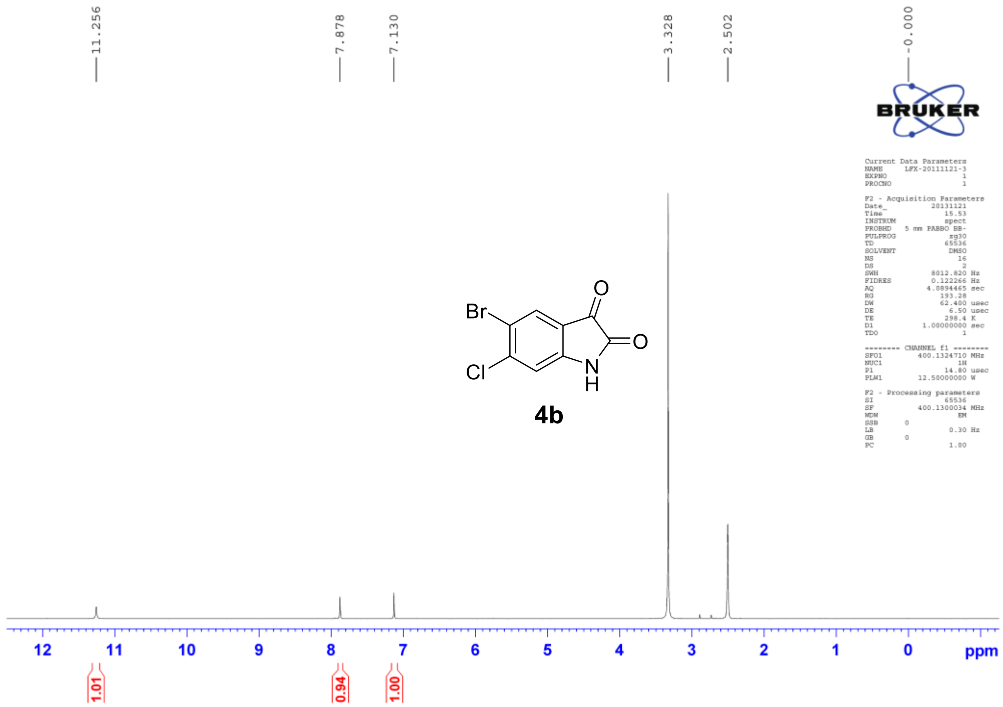
11.256
7.878
7.130
3.328
2.502
-0.000
BRUKER
Br
Cl
O
O
N
H
4b
12 11 10 9 8 7 6 5 4 3 2 1 0 ppm
1.01
0.94
1.00

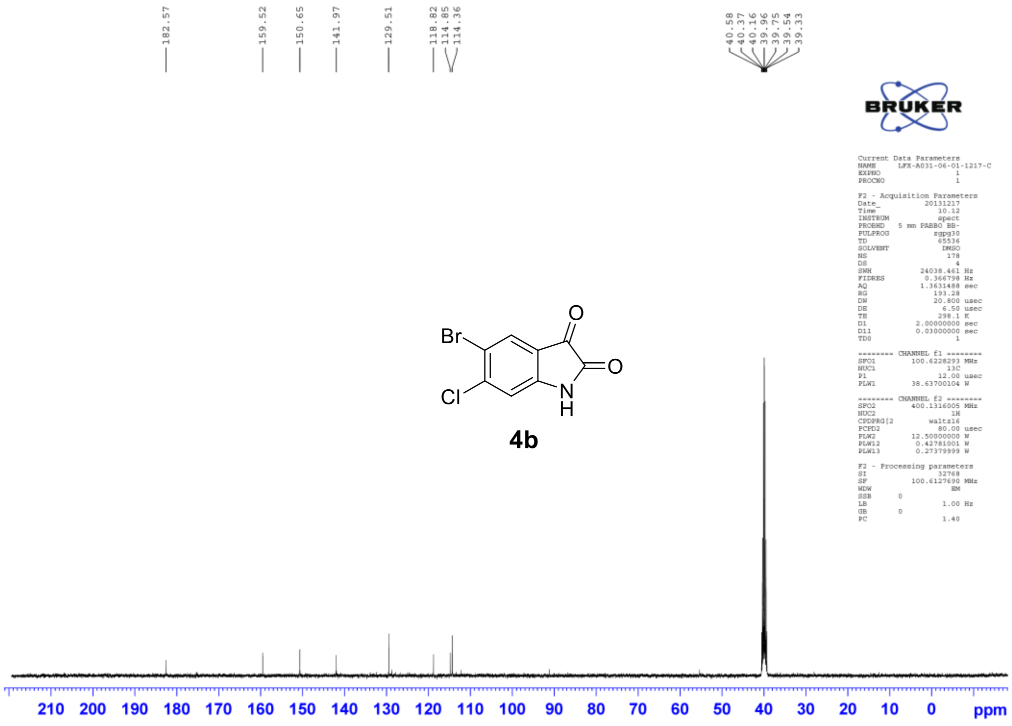
182.57
159.52
150.65
141.97
129.51
118.82
114.85
114.36
40.58
40.37
40.16
39.96
39.75
39.54
39.33
BRUKER
Br
Cl
O
O
N
H
4b
210 200 190 180 170 160 150 140 130 120 110 100 90 80 70 60 50 40 30 20 10 0 ppm

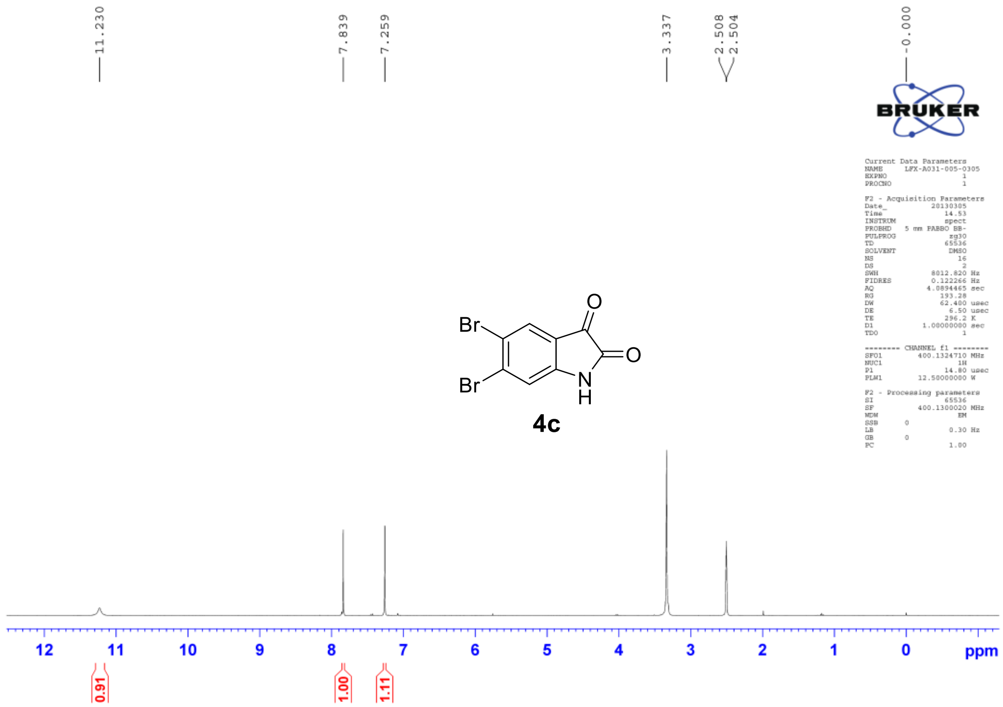
11.230
7.839
7.259
3.337
2.508
2.504
-0.000
BRUKER
Br
Br
O
O
N
H
4c
12 11 10 9 8 7 6 5 4 3 2 1 0 ppm
0.91
1.00
1.11

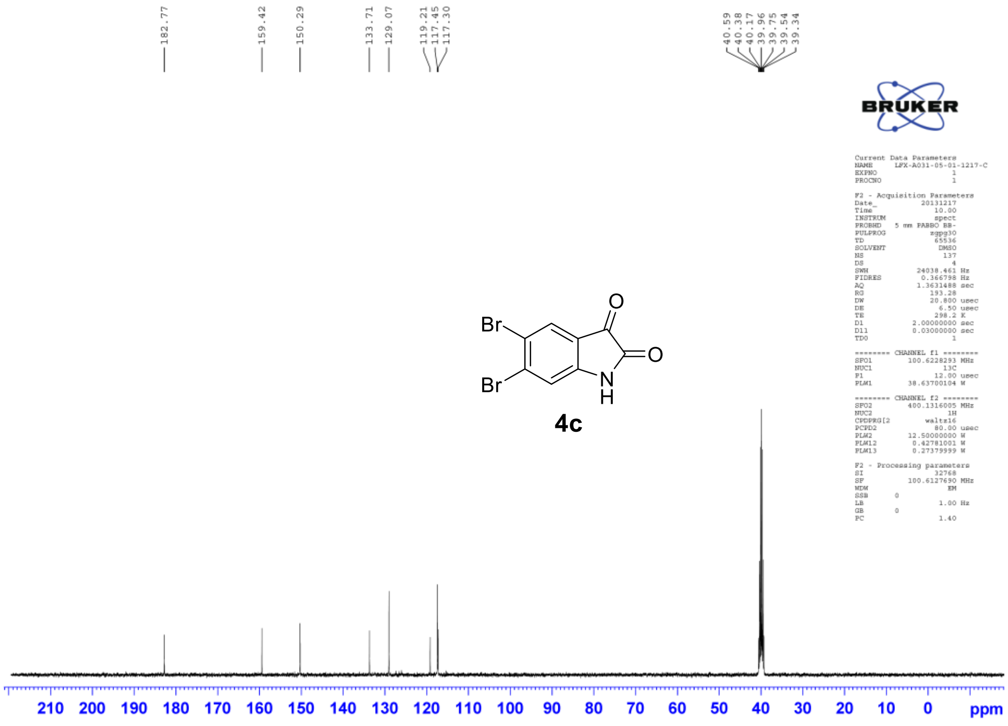
182.77
159.42
150.29
133.71
129.07
119.21
117.45
117.30
40.59
40.38
40.17
39.96
39.75
39.54
39.34
BRUKER
Br
Br
O
O
N
H
4c
210 200 190 180 170 160 150 140 130 120 110 100 90 80 70 60 50 40 30 20 10 0 ppm

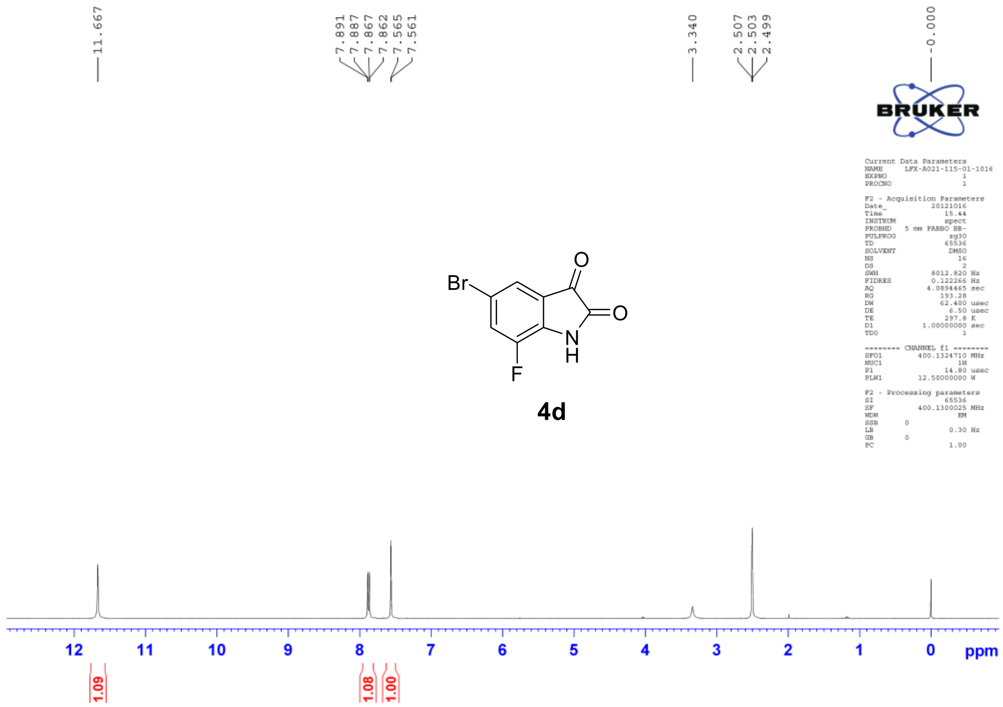
11.667
7.891
7.887
7.867
7.862
7.565
7.561
3.340
2.507
2.503
2.499
0.000
BRUKER
Br
O
O
N
H
F
4d
12 11 10 9 8 7 6 5 4 3 2 1 0 ppm
1.09
1.08
1.00

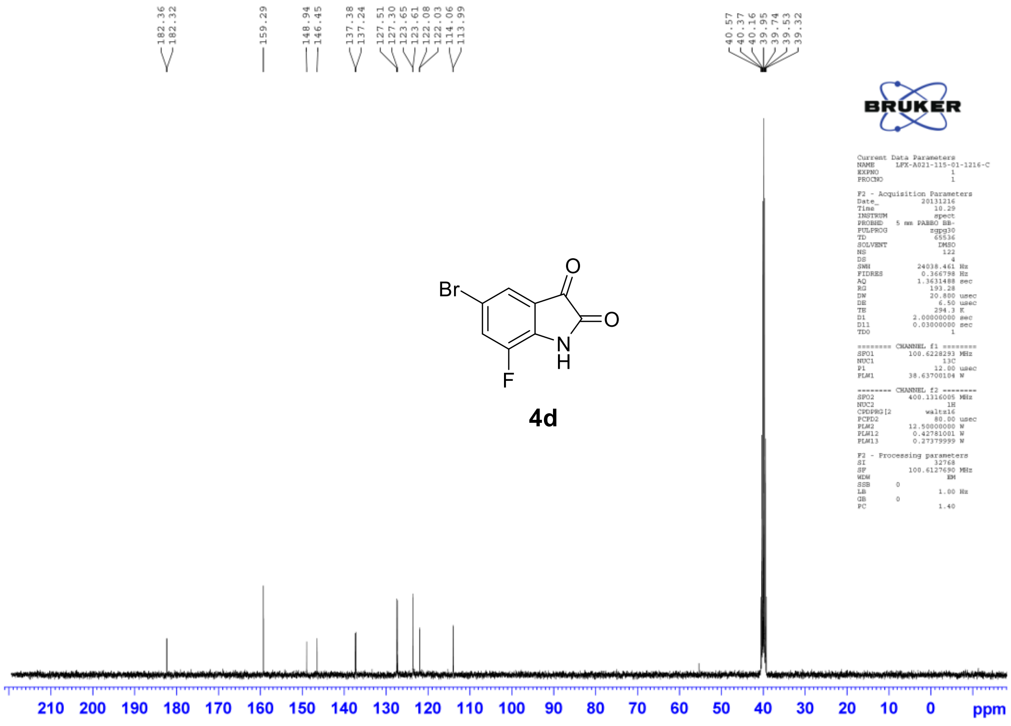
182.36
182.32
159.29
148.94
146.45
137.38
137.24
127.51
127.30
123.65
123.61
122.08
122.03
114.06
113.99
40.57
40.37
40.16
39.95
39.74
39.53
39.32
BRUKER
Br
O
O
N
H
F
4d
210 200 190 180 170 160 150 140 130 120 110 100 90 80 70 60 50 40 30 20 10 0 ppm

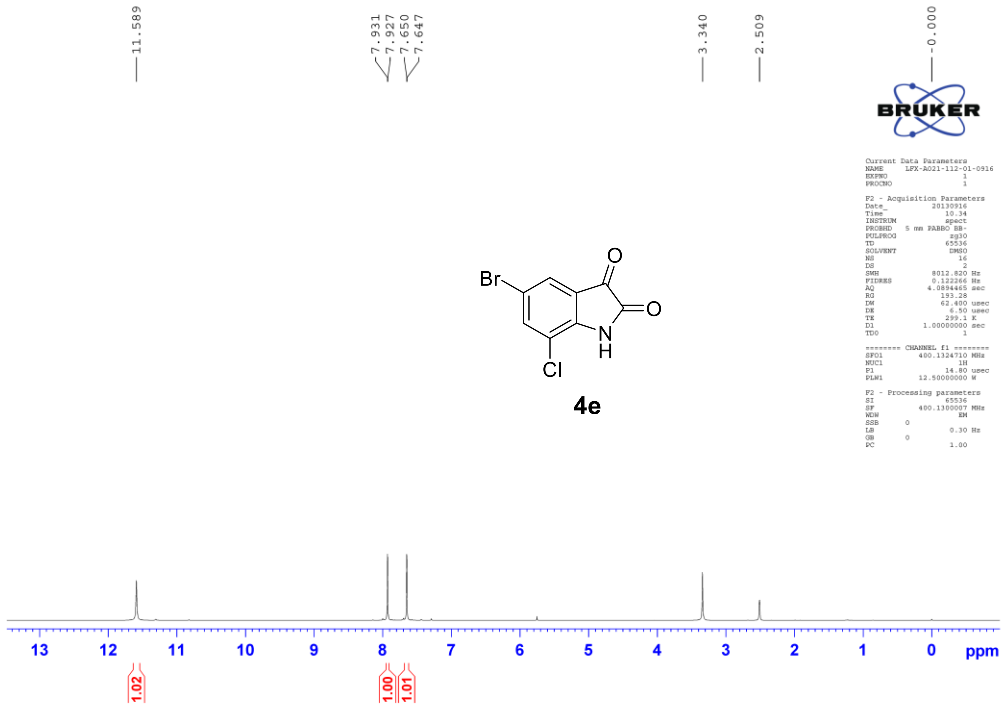
11.589
7.931
7.927
7.650
7.647
3.340
2.509
-0.000
BRUKER
Br
O
O
N
H
Cl
4e
13 12 11 10 9 8 7 6 5 4 3 2 1 0 ppm
1.02
1.00
1.01

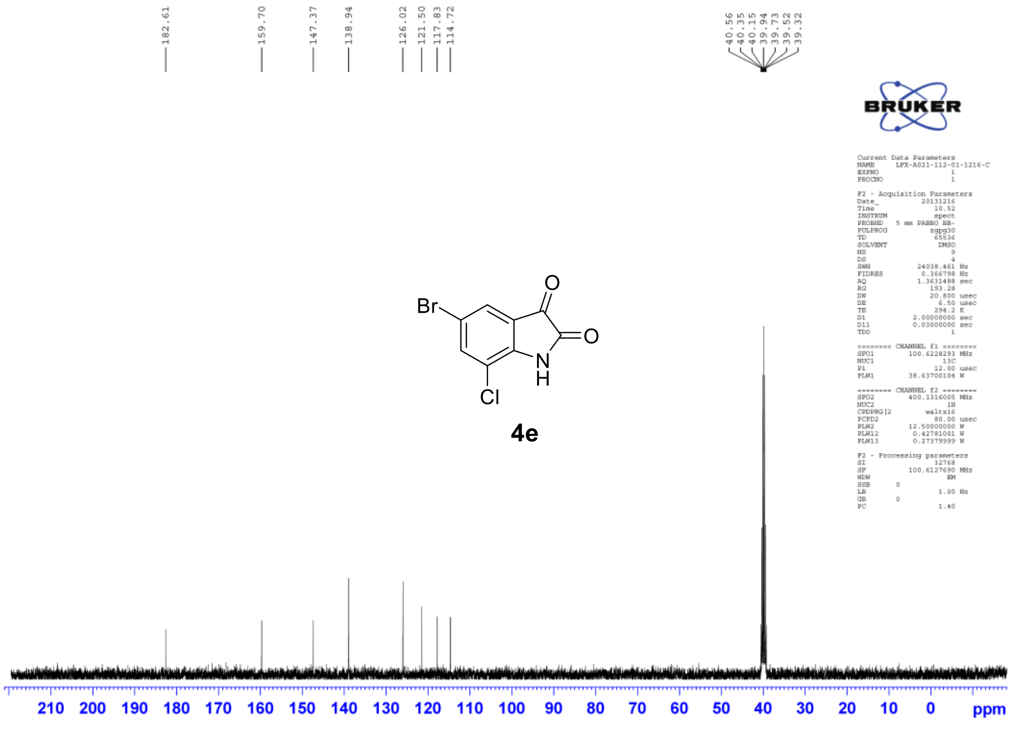
182.61
159.70
147.37
138.94
126.02
121.50
117.83
114.72
40.56
40.35
40.15
39.94
39.73
39.52
39.32
BRUKER
Br
O
O
N
H
Cl
4e
210 200 190 180 170 160 150 140 130 120 110 100 90 80 70 60 50 40 30 20 10 0 ppm

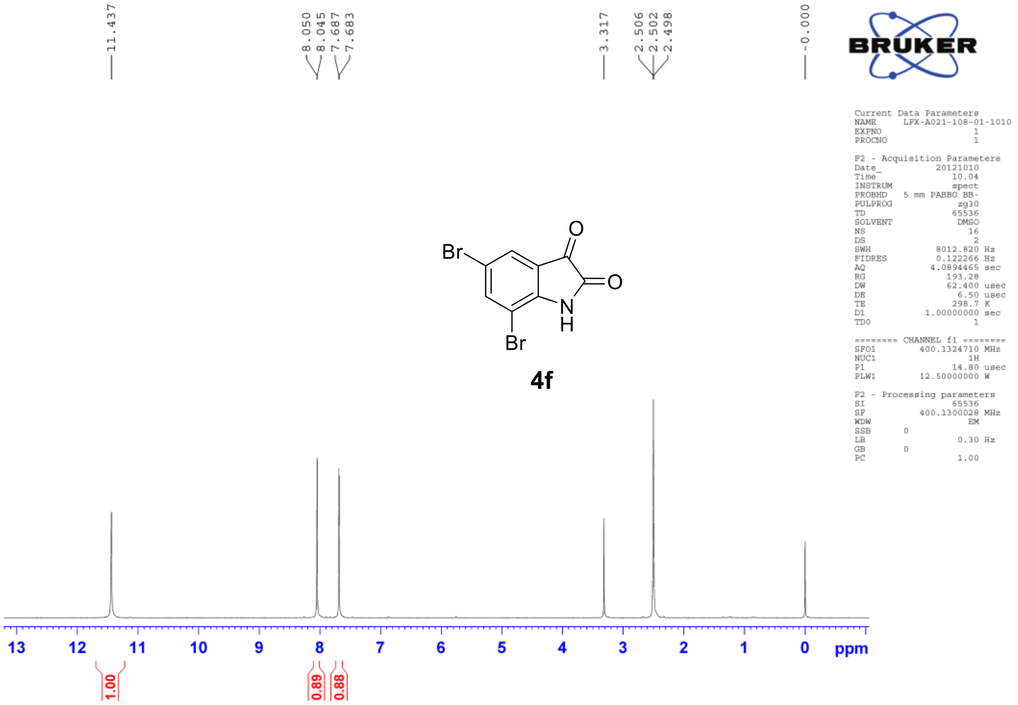
11.437
8.050
8.045
7.687
7.683
3.317
2.506
2.502
2.498
-0.000
BRUKER
4f
13 12 11 10 9 8 7 6 5 4 3 2 1 0 ppm
1.00
0.89
0.88

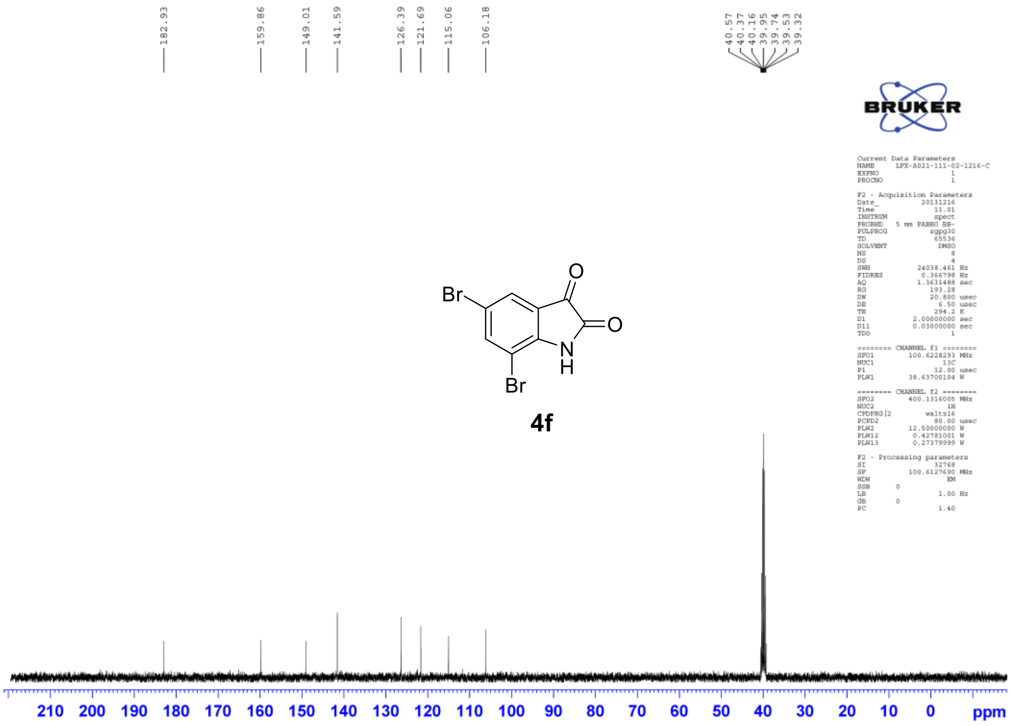
182.93
159.86
149.01
141.59
126.39
121.69
115.06
106.18
40.57
40.37
40.16
39.95
39.74
39.53
39.32
BRUKER
4f
210 200 190 180 170 160 150 140 130 120 110 100 90 80 70 60 50 40 30 20 10 0 ppm

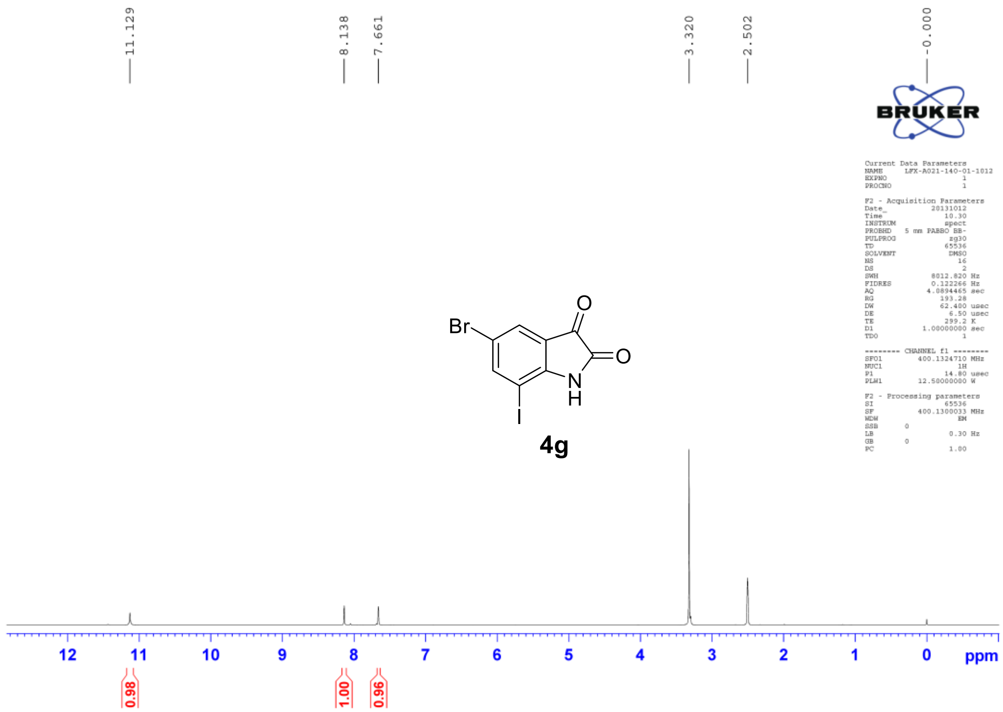
11.129
8.138
7.661
3.320
2.502
-0.000
BRUKER
Br
O
O
N
H
I
4g
12 11 10 9 8 7 6 5 4 3 2 1 0 ppm
0.98
1.00
0.96

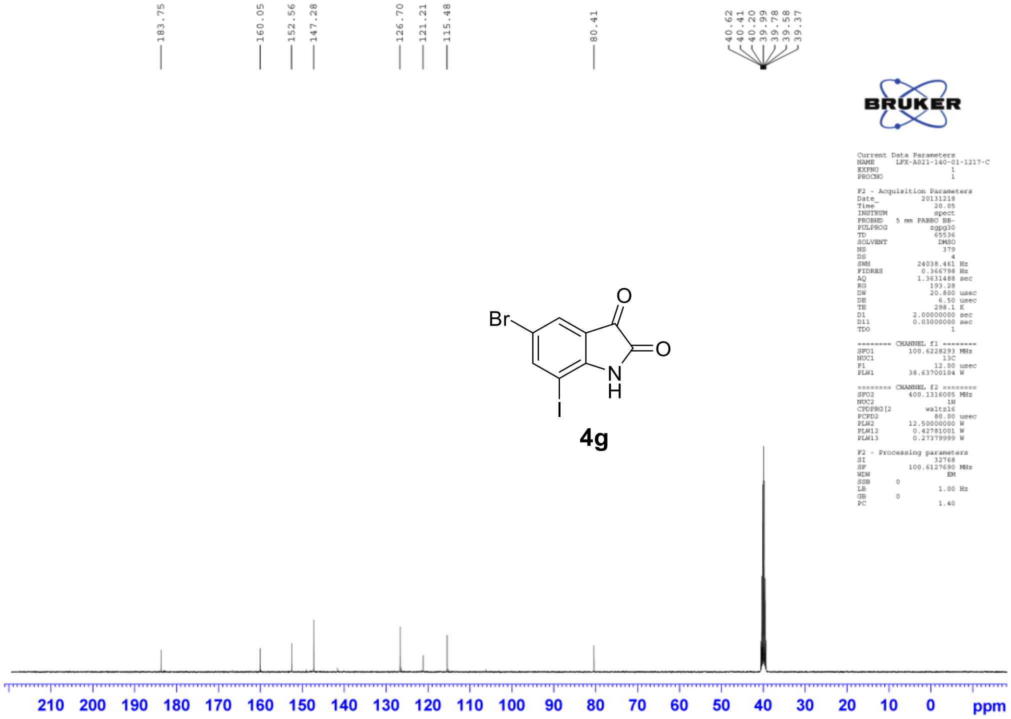
183.75
160.05
152.56
147.28
126.70
121.21
115.48
80.41
40.62
40.41
40.20
39.99
39.78
39.58
39.37
BRUKER
Br
O
O
N
H
I
4g
210 200 190 180 170 160 150 140 130 120 110 100 90 80 70 60 50 40 30 20 10 0 ppm

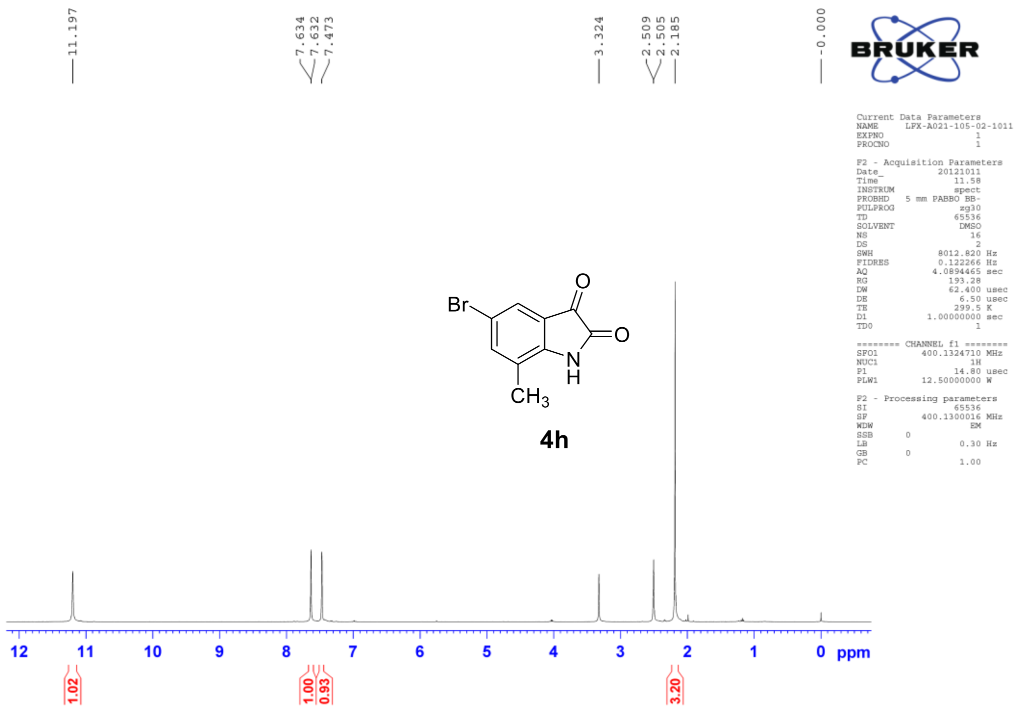
11.197
7.634
7.632
7.473
3.324
2.509
2.505
2.185
-0.000
BRUKER
Br
O
O
N
H
CH3
4h
1.02
1.00
0.93
3.20
ppm

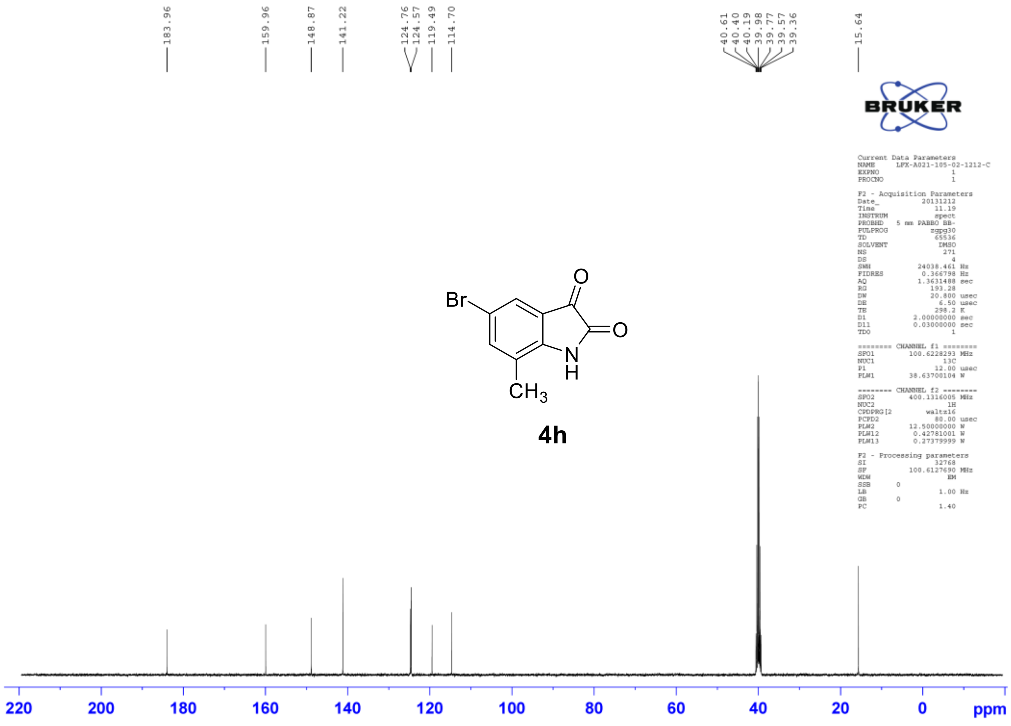
183.96
159.96
148.87
141.22
124.76
124.57
119.49
114.70
40.61
40.40
40.19
39.98
39.77
39.57
39.36
15.64
BRUKER
Br
O
O
N
H
CH3
4h
ppm

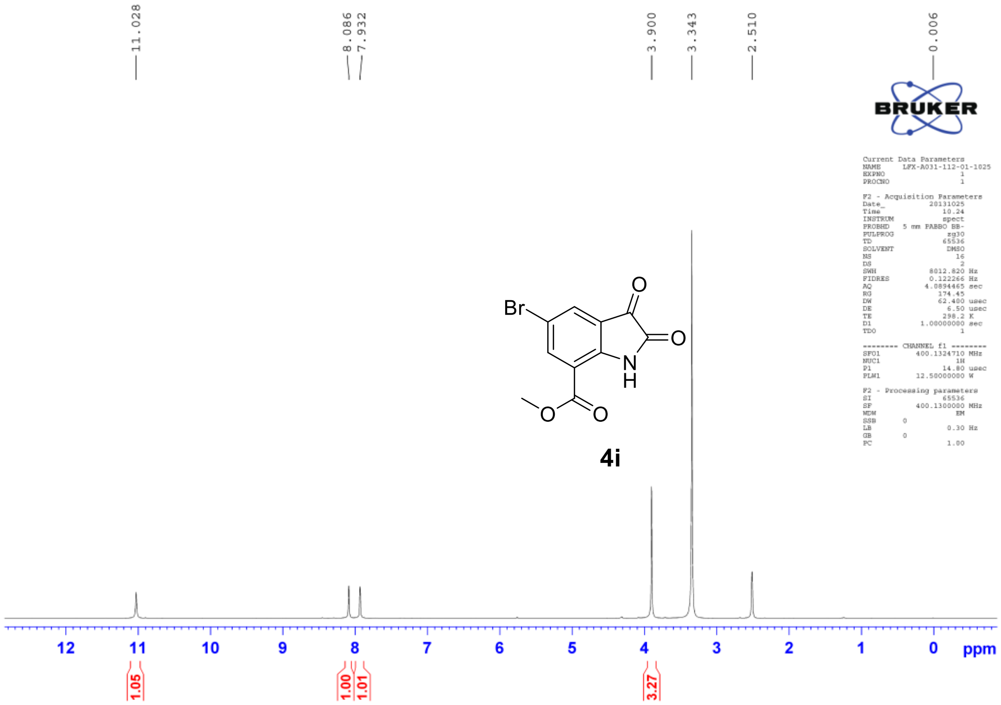
11.028
8.086
7.932
3.900
3.343
2.510
0.006
BRUKER
Br
O
O
NH
O
O
4i
12 11 10 9 8 7 6 5 4 3 2 1 0 ppm
1.05
1.00
1.01
3.27

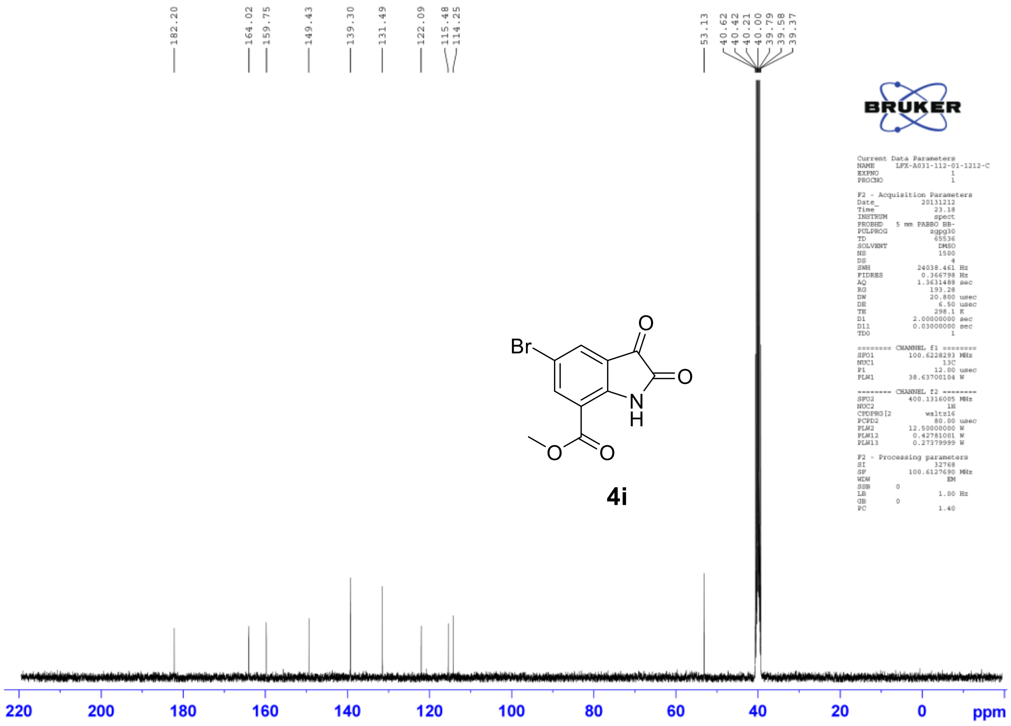
182.20
164.02
159.75
149.43
139.30
131.49
122.09
115.48
114.25
53.13
40.62
40.42
40.21
40.00
39.79
39.58
39.37
BRUKER
Br
O
O
NH
O
O
4i
220 200 180 160 140 120 100 80 60 40 20 0 ppm

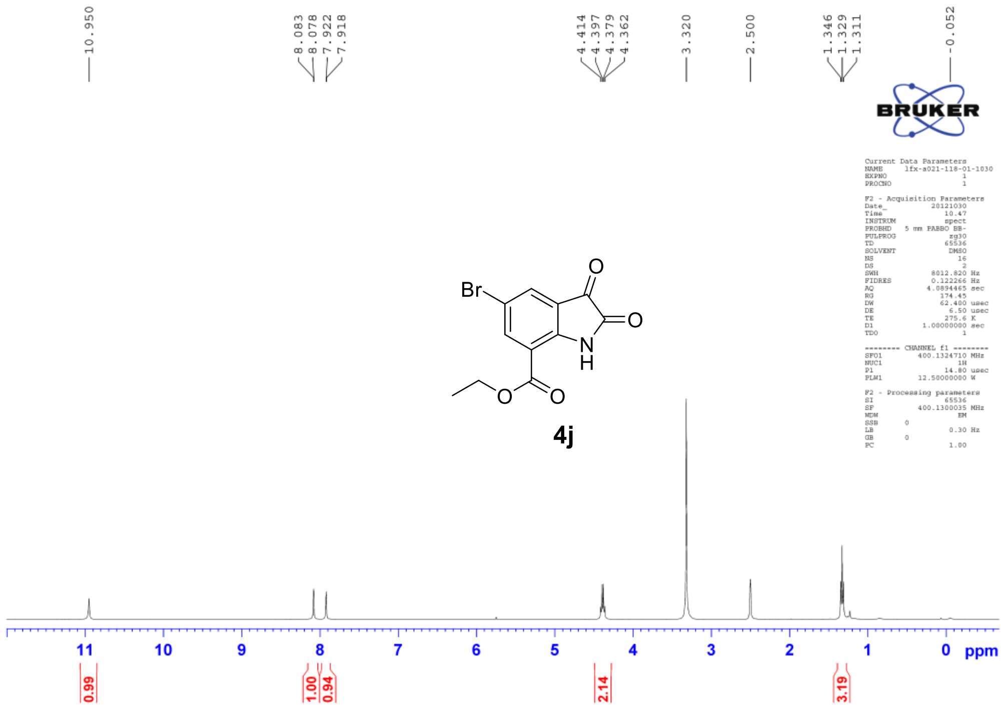
10.950
8.083
8.078
7.922
7.918
4.414
4.397
4.379
4.362
3.320
2.500
1.346
1.329
1.311
-0.052
Br
O
O
N
H
O
O
4j
11 10 9 8 7 6 5 4 3 2 1 0 ppm
0.99
1.00
0.94
2.14
3.19

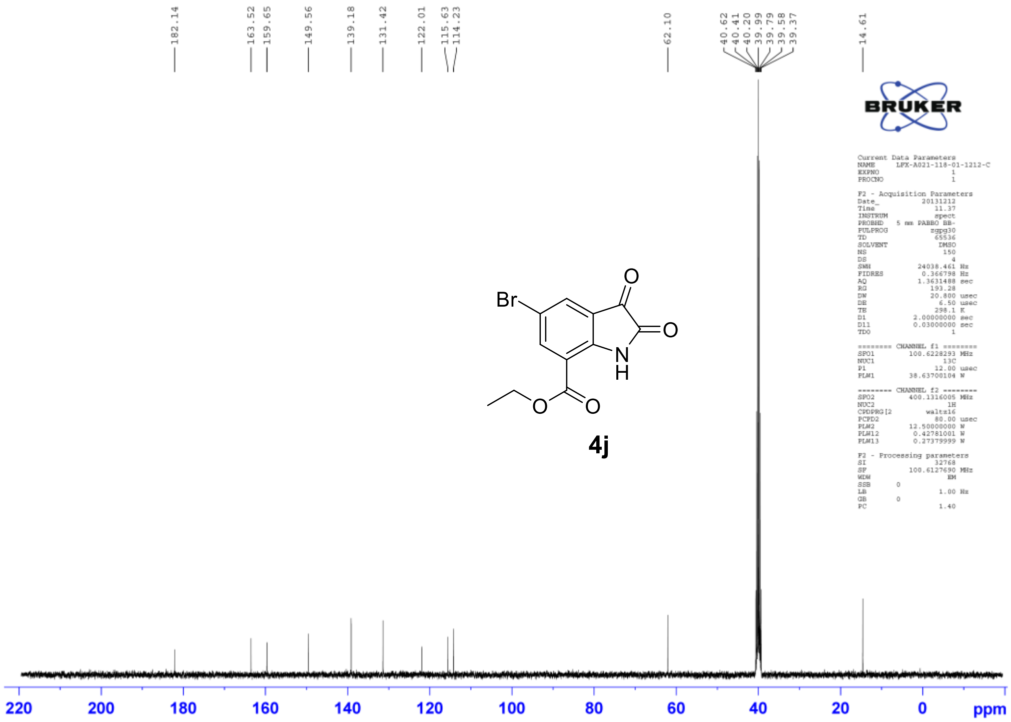
182.14
163.52
159.65
149.56
139.18
131.42
122.01
115.63
114.23
62.10
40.62
40.41
40.20
39.99
39.79
39.58
39.37
14.61
Br
O
O
N
H
O
O
4j
220 200 180 160 140 120 100 80 60 40 20 0 ppm

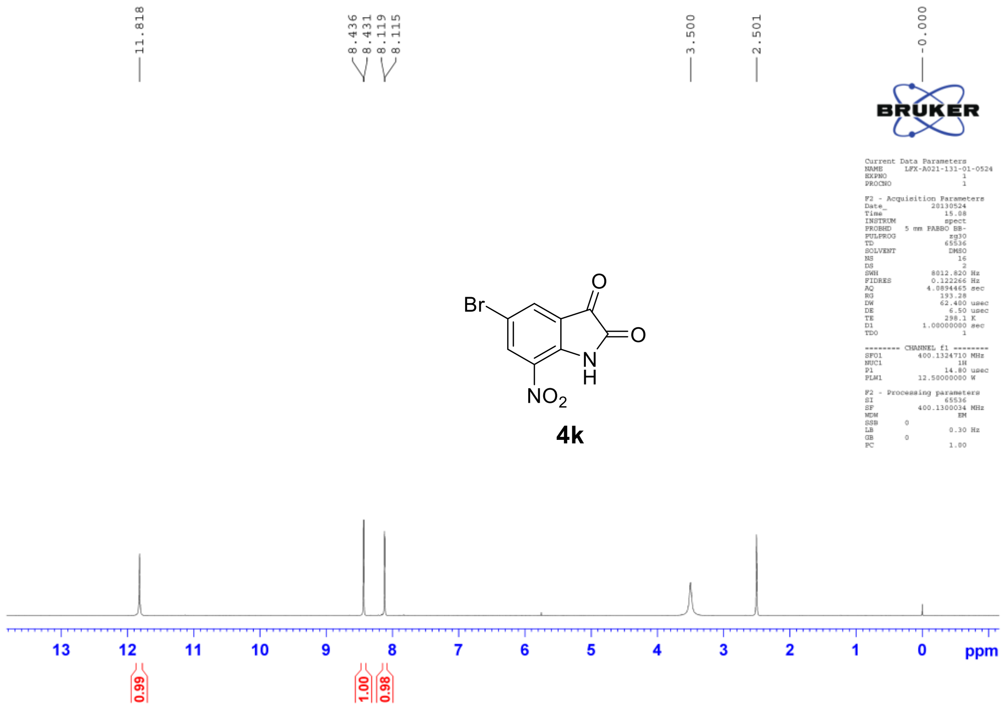
11.818
8.436
8.431
8.119
8.115
3.500
2.501
-0.000
BRUKER
Br
O
O
N
H
NO2
4k
13
12
11
10
9
8
7
6
5
4
3
2
1
0
ppm
0.99
1.00
0.98

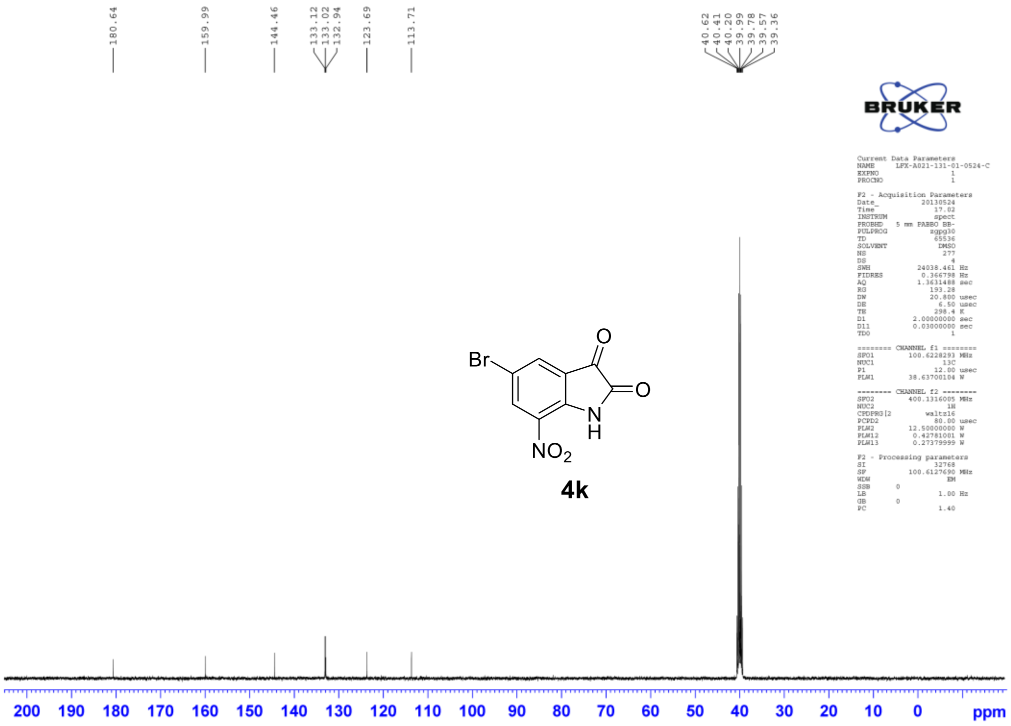
180.64
159.99
144.46
133.12
133.02
132.94
123.69
113.71
40.62
40.41
40.20
39.99
39.78
39.57
39.36
BRUKER
Br
O
O
N
H
NO2
4k
200
190
180
170
160
150
140
130
120
110
100
90
80
70
60
50
40
30
20
10
0
ppm

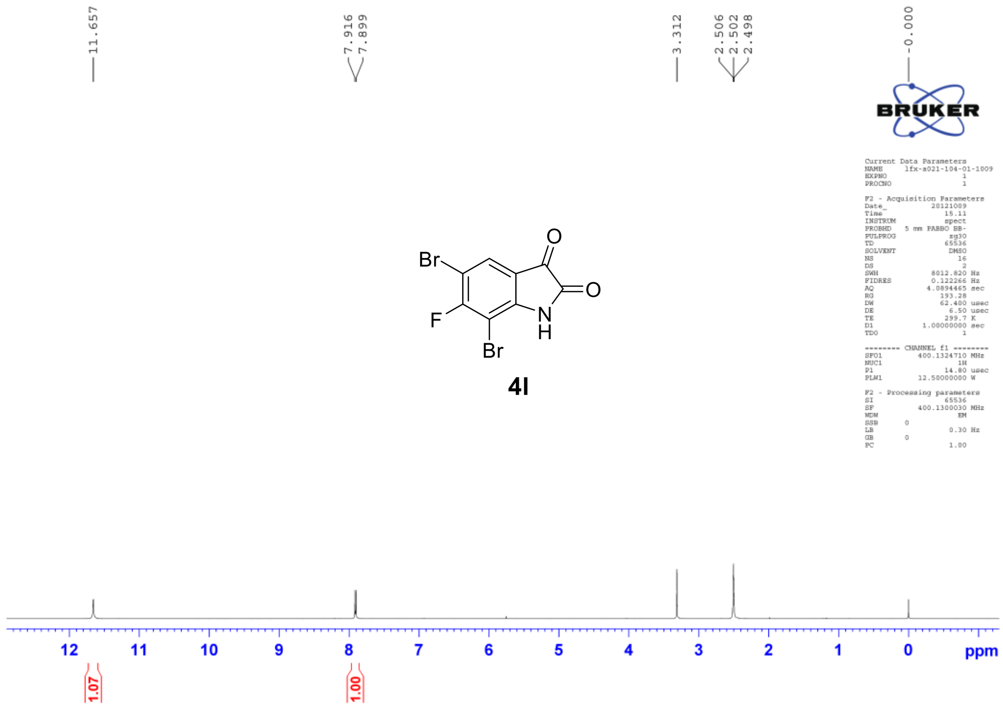
11.657
7.916
7.899
3.312
2.506
2.502
2.498
-0.000
BRUKER
4l
12 11 10 9 8 7 6 5 4 3 2 1 0 ppm
1.07
1.00

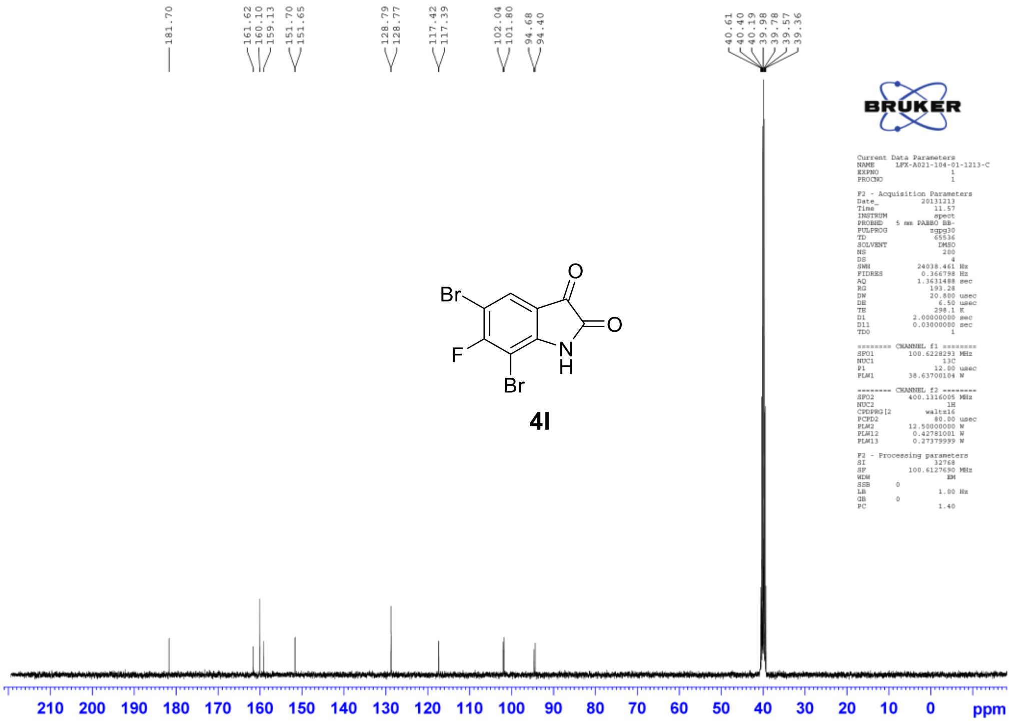
181.70
161.62
160.10
159.13
151.70
151.65
128.79
128.77
117.42
117.39
102.04
101.80
94.68
94.40
40.61
40.40
40.19
39.98
39.78
39.57
39.36
BRUKER
4l
210 200 190 180 170 160 150 140 130 120 110 100 90 80 70 60 50 40 30 20 10 0 ppm

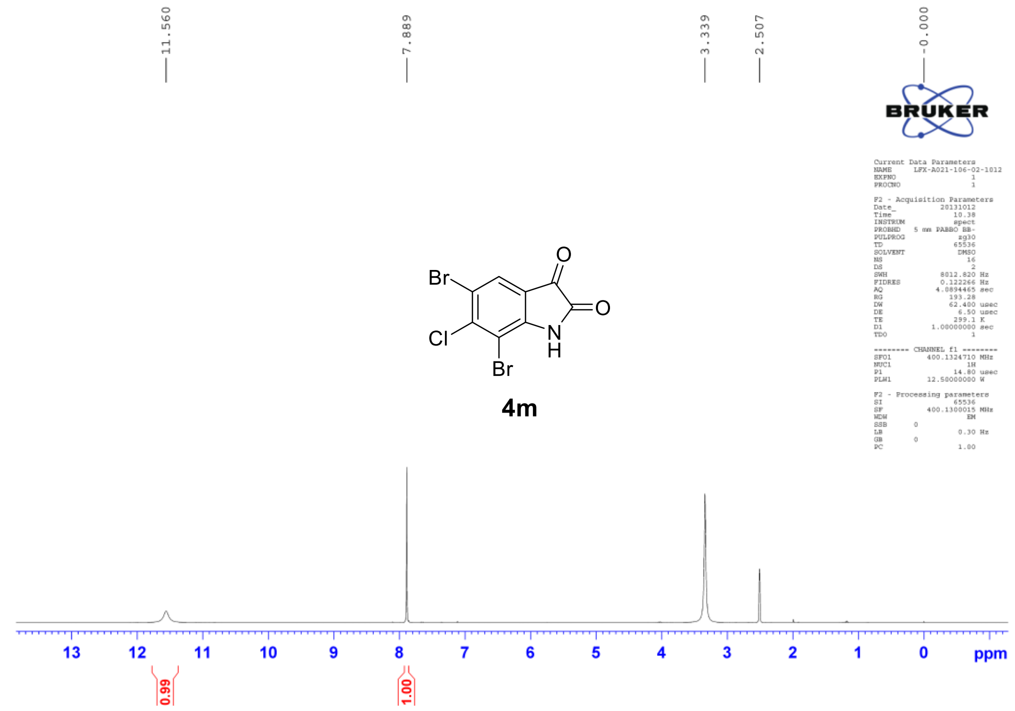
11.560
7.889
3.339
2.507
-0.000
Br
Cl
Br
O
O
N
H
4m
13 12 11 10 9 8 7 6 5 4 3 2 1 0 ppm
0.99
1.00

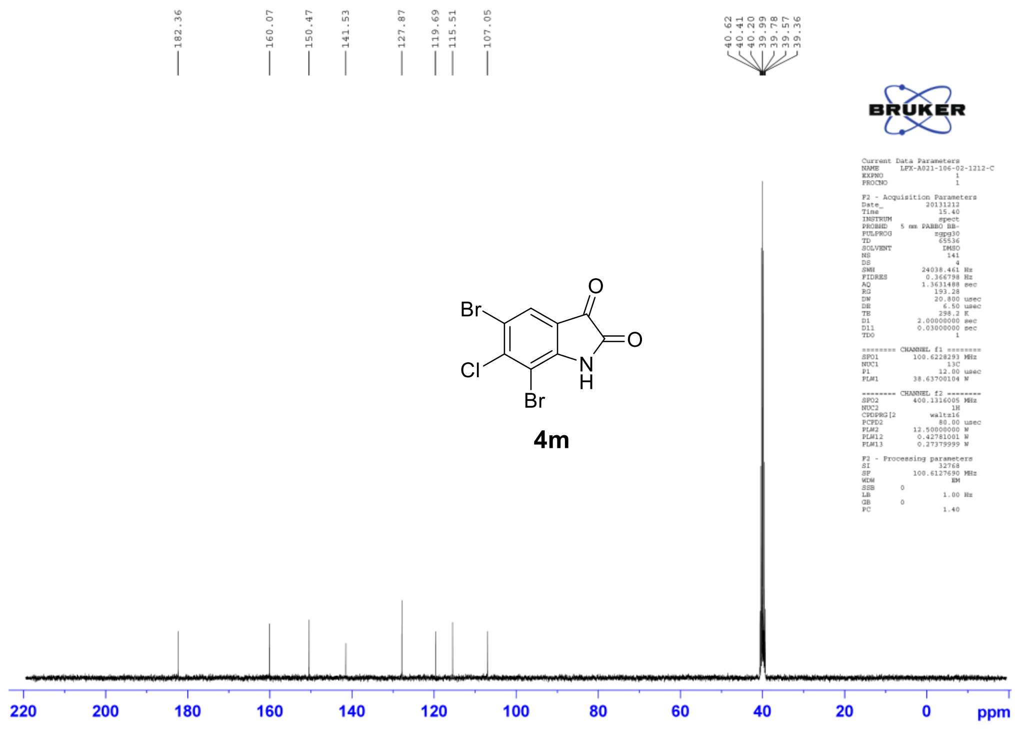
182.36
160.07
150.47
141.53
127.87
119.69
115.51
107.05
40.62
40.41
40.20
39.99
39.78
39.57
39.36
Br
Cl
Br
O
O
N
H
4m
220 200 180 160 140 120 100 80 60 40 20 0 ppm

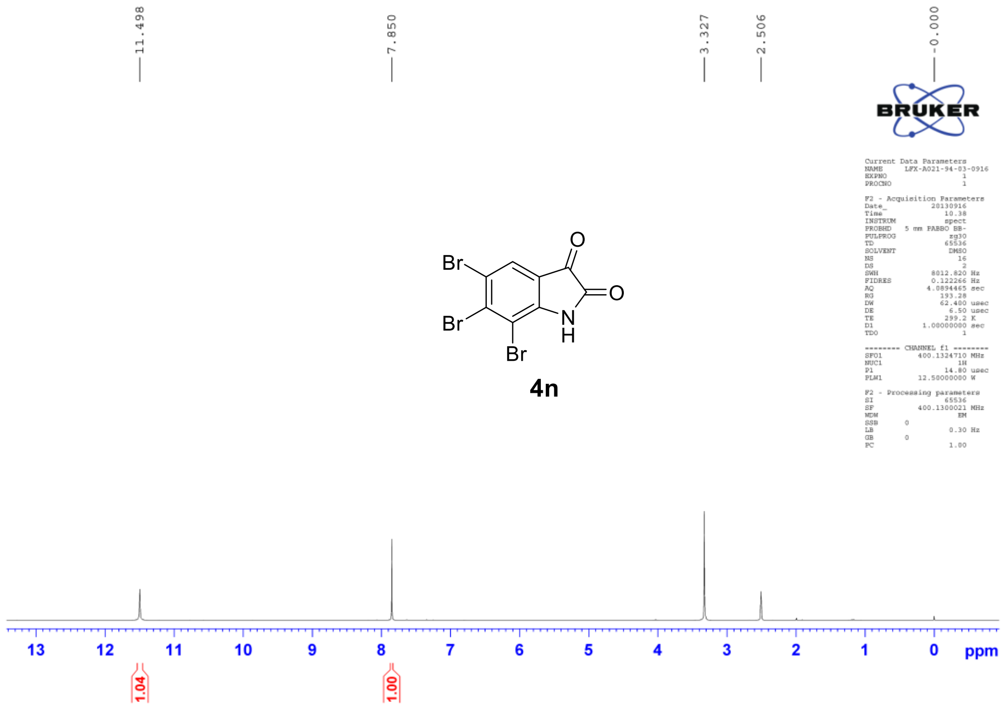
11.498
7.850
3.327
2.506
-0.000
BRUKER
Br
Br
Br
O
O
N
H
4n
1.04
1.00
13 12 11 10 9 8 7 6 5 4 3 2 1 0 ppm

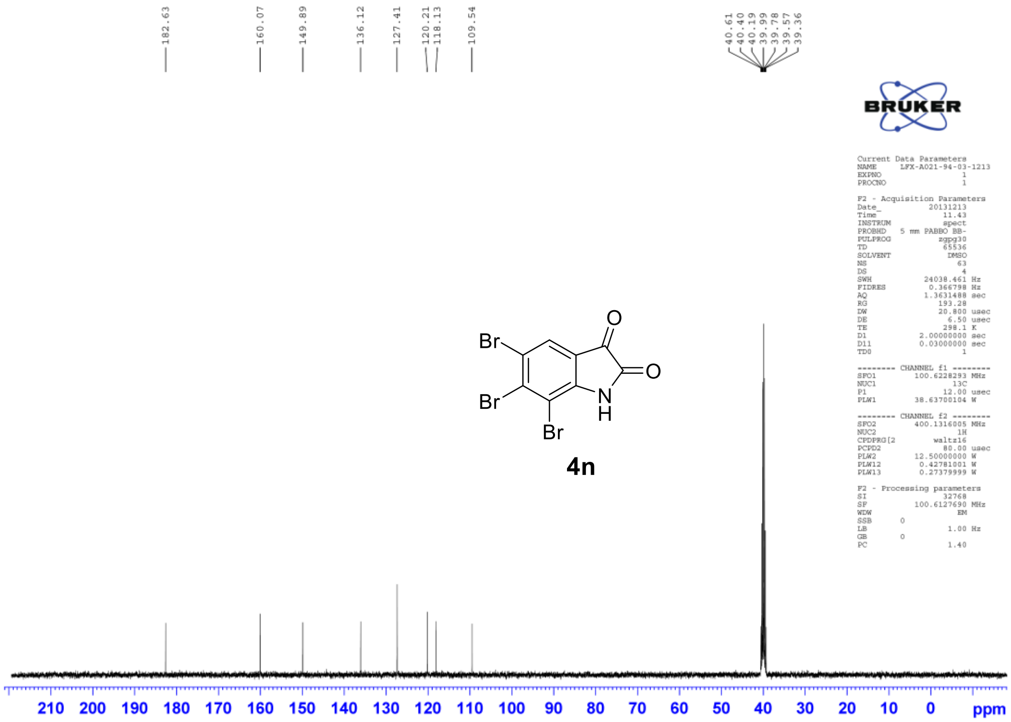
182.63
160.07
149.89
136.12
127.41
120.21
118.13
109.54
40.61
40.40
40.19
39.99
39.78
39.57
39.36
BRUKER
Br
Br
Br
O
O
N
H
4n
210 200 190 180 170 160 150 140 130 120 110 100 90 80 70 60 50 40 30 20 10 0 ppm

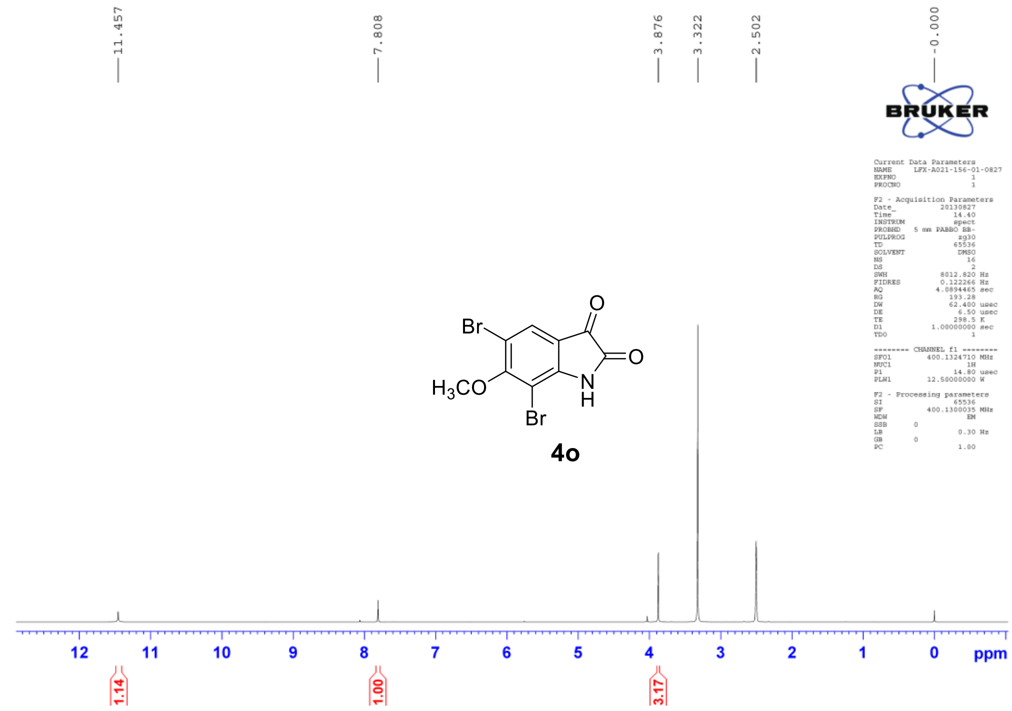
11.457
7.808
3.876
3.322
2.502
-0.000
BRUKER
Br
O
O
$H_3CO$
NH
Br
4o
12 11 10 9 8 7 6 5 4 3 2 1 0 ppm
1.14
1.00
3.17

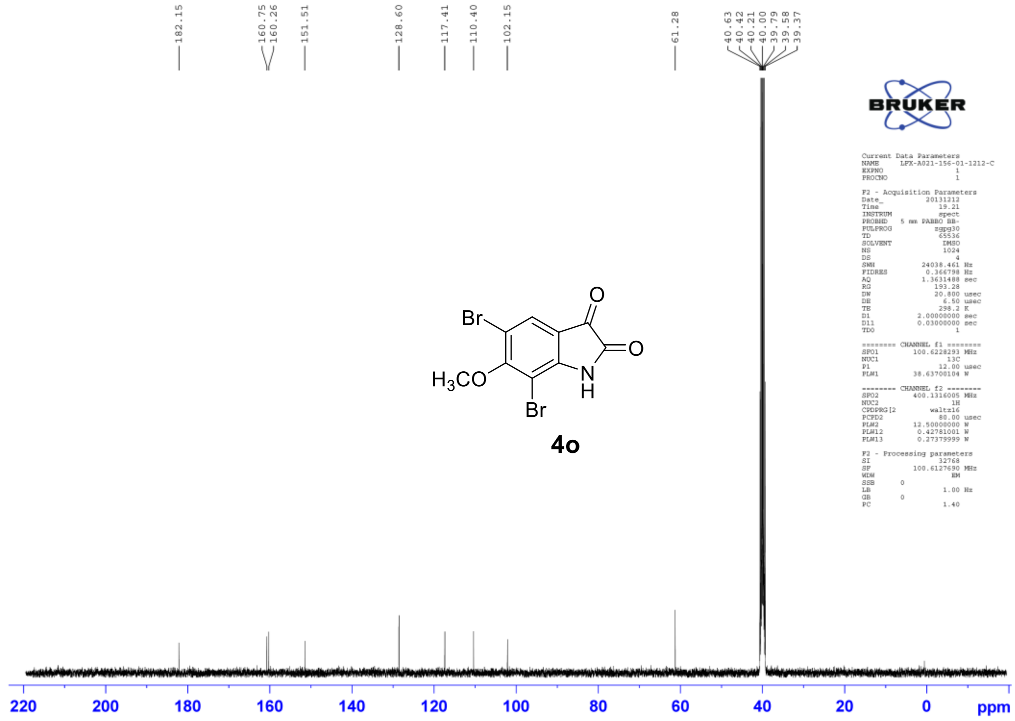
182.15
160.75
160.26
151.51
128.60
117.41
110.40
102.15
61.28
40.63
40.42
40.21
40.00
39.79
39.58
39.37
BRUKER
Br
O
O
$H_3CO$
NH
Br
4o
220 200 180 160 140 120 100 80 60 40 20 0 ppm

### 3. HR-MS Spectrum Data of compound 4l,4m,4o.

PDF Complete
Your complimentary use period has ended. Thank you for using PDF Complete.
Click Here to upgrade to Unlimited Pages and Expanded Features



| [illegible] | [illegible] | [illegible] | [illegible] | [illegible] | [illegible] | [illegible] | [illegible] | [illegible]t | Val. | Min | Max | Elmt | Val. | Min | Max | Use Adduct |
|---|---|---|---|---|---|---|---|---|---|---|---|---|---|---|---|---|
| [illegible] | [illegible] | [illegible] | [illegible] | [illegible] | [illegible] | [illegible] | [illegible] | [illegible] | 2 | 0 | 0 | Se | 2 | 0 | 0 | H |
| 2H | 1 | 0 | 0 | F | 1 | 0 | 1 | Cl | 1 | 0 | 0 | Br | 1 | 0 | 2 | HCOO |
| C | 4 | 0 | 8 | Na | 1 | 0 | 0 | K | 1 | 0 | 0 | I | 3 | 0 | 0 | CH3COO |
| N | 3 | 0 | 1 | Si | 4 | 0 | 0 | Cr | 2 | 0 | 0 | Pt | 2 | 0 | 0 | Cl |
| O | 2 | 0 | 2 | P | 3 | 0 | 0 | Ni | 2 | 0 | 0 | | | | | |

| | | | | | |
|---|---|---|---|---|---|
| Error Margin (ppm): | 9999 | DBE Range: | 0.0 - 50.0 | Electron Ions: | both |
| HC Ratio: | unlimited | Apply N Rule: | no | Use MSn Info: | yes |
| Max Isotopes: | all | Isotope RI (%): | 1.00 | Isotope Res: | 10000 |
| MSn Iso RI (%): | 75.00 | MSn Logic Mode: | AND | Max Results: | 100 |

Event#: 2 MS(E-) Ret. Time : 1.187 Scan# : 180

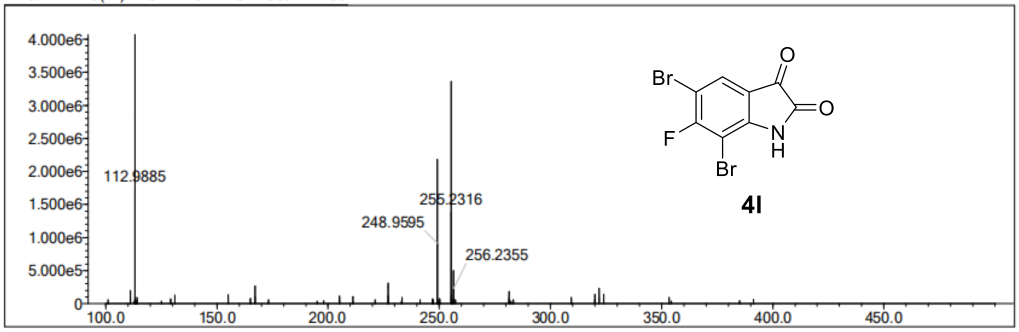


Measured region for 319.8339 m/z

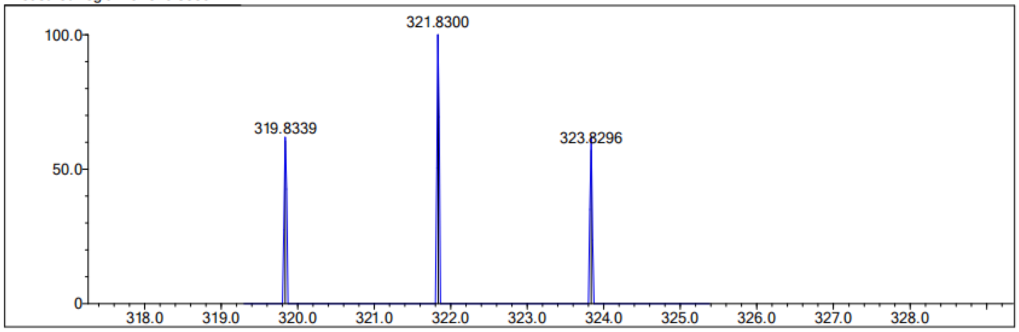


C8 H2 N O2 F Br2 [M-H]- : Predicted region for 319.8364 m/z

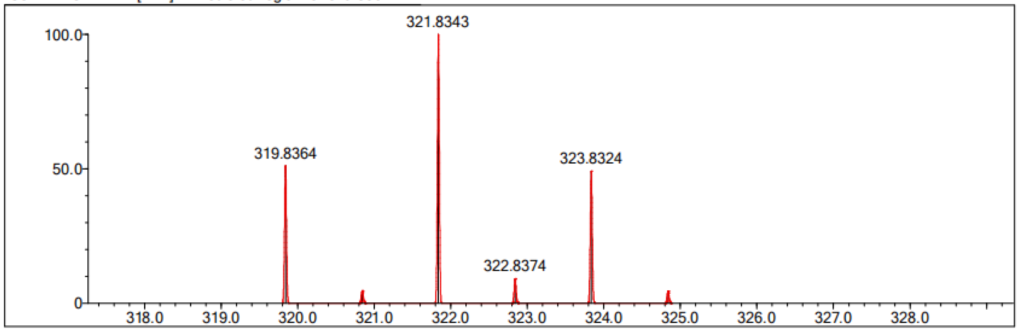


| Rank | Score | Formula (M) | Ion | Meas. m/z | Pred. m/z | Df. (mDa) | Df. (ppm) | Iso | DBE |
|---|---|---|---|---|---|---|---|---|---|
| 2 | 28.98 | C8 H2 N O2 F Br2 | [M-H]- | 319.8339 | 319.8364 | -2.5 | -7.82 | 46.89 | 7.0 |

PDF Complete
Your complimentary use period has ended. Thank you for using PDF Complete.
Click Here to upgrade to Unlimited Pages and Expanded Features



| [illegible] | [illegible] | [illegible] | [illegible] | [illegible] | [illegible] | [illegible] | [illegible] | [illegible]t | Val. | Min | Max | Elmt | Val. | Min | Max | Use Adduct |
|---|---|---|---|---|---|---|---|---|---|---|---|---|---|---|---|---|
| [illegible] | [illegible] | [illegible] | [illegible] | [illegible] | [illegible] | [illegible] | [illegible] | [illegible] | 2 | 0 | 0 | Se | 2 | 0 | 0 | H |
| 2H | 1 | 0 | 0 | F | 1 | 0 | 0 | Cl | 1 | 0 | 0 | Br | 1 | 0 | 2 | HCOO |
| C | 4 | 0 | 9 | Na | 1 | 0 | 0 | K | 1 | 0 | 0 | I | 3 | 0 | 0 | CH3COO |
| N | 3 | 0 | 1 | Si | 4 | 0 | 0 | Cr | 2 | 0 | 0 | Pt | 2 | 0 | 0 | Cl |
| O | 2 | 0 | 3 | P | 3 | 0 | 0 | Ni | 2 | 0 | 0 | | | | | |

| | | | | | |
|---|---|---|---|---|---|
| Error Margin (ppm): | 9999 | DBE Range: | 0.0 - 50.0 | Electron Ions: | both |
| HC Ratio: | unlimited | Apply N Rule: | no | Use MSn Info: | yes |
| Max Isotopes: | all | Isotope RI (%): | 1.00 | Isotope Res: | 10000 |
| MSn Iso RI (%): | 75.00 | MSn Logic Mode: | AND | Max Results: | 100 |

Event#: 2 MS(E-) Ret. Time : 1.120 Scan# : 170

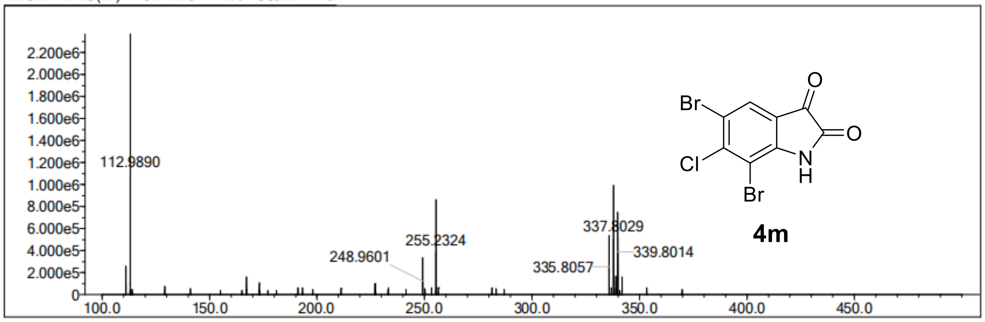


**4m**

Measured region for 335.8049 m/z

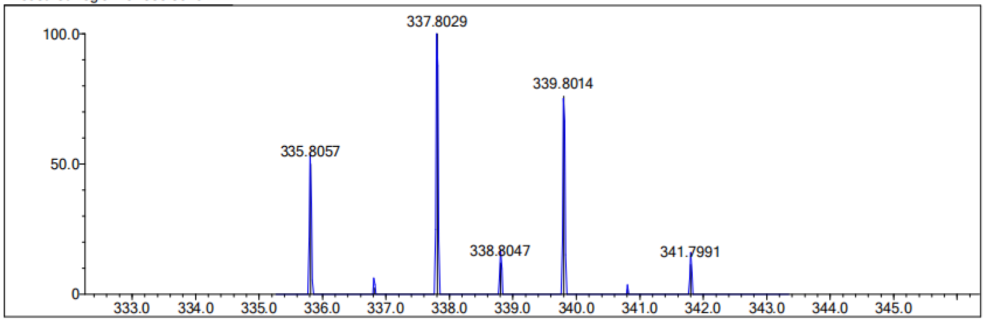


C8 H N O2 Br2 [M+Cl]- : Predicted region for 335.8068 m/z

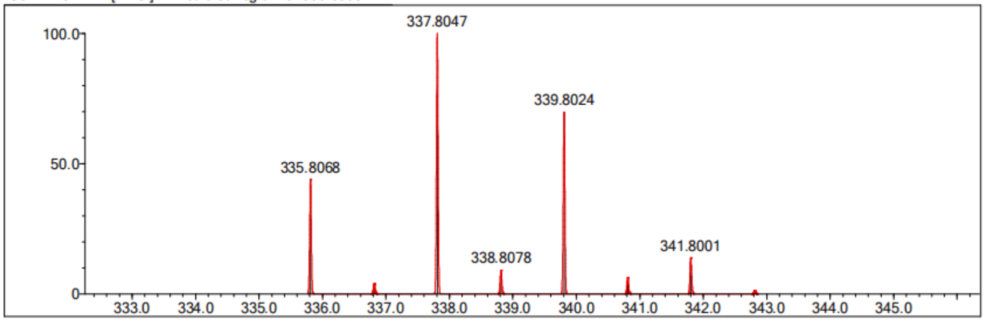


| Rank | Score | Formula (M) | Ion | Meas. m/z | Pred. m/z | Df. (mDa) | Df. (ppm) | Iso | DBE |
|---|---|---|---|---|---|---|---|---|---|
| 1 | 70.53 | C8 H N O2 Br2 | [M+Cl]- | 335.8049 | 335.8068 | -1.9 | -5.66 | 84.57 | 8.0 |

PDF Complete
Your complimentary use period has ended. Thank you for using PDF Complete.
Click Here to upgrade to Unlimited Pages and Expanded Features



| [illegible] | [illegible] | [illegible] | [illegible] | [illegible] | [illegible] | [illegible] | [illegible] | [illegible] | Val. | Min | Max | Elmt | Val. | Min | Max | Use Adduct |
|---|---|---|---|---|---|---|---|---|---|---|---|---|---|---|---|---|
| [illegible] | [illegible] | [illegible] | [illegible] | [illegible] | [illegible] | [illegible] | [illegible] | [illegible] | 2 | 0 | 0 | Se | 2 | 0 | 0 | H |
| 2H | 1 | 0 | 0 | F | 1 | 0 | 0 | Cl | 1 | 0 | 0 | Br | 1 | 0 | 2 | HCOO |
| C | 4 | 0 | 9 | Na | 1 | 0 | 0 | K | 1 | 0 | 0 | I | 3 | 0 | 0 | CH3COO |
| N | 3 | 0 | 1 | Si | 4 | 0 | 0 | Cr | 2 | 0 | 0 | Pt | 2 | 0 | 0 | Cl |
| O | 2 | 0 | 3 | P | 3 | 0 | 0 | Ni | 2 | 0 | 0 | | | | | |

| | | | | | |
|---|---|---|---|---|---|
| Error Margin (ppm): | 9999 | DBE Range: | 0.0 - 50.0 | Electron Ions: | both |
| HC Ratio: | unlimited | Apply N Rule: | no | Use MSn Info: | yes |
| Max Isotopes: | all | Isotope RI (%): | 1.00 | Isotope Res: | 10000 |
| MSn Iso RI (%): | 75.00 | MSn Logic Mode: | AND | Max Results: | 100 |

Event#: 2 MS(E-) Ret. Time : 1.067 -> 1.067 Scan# : 162 -> 162

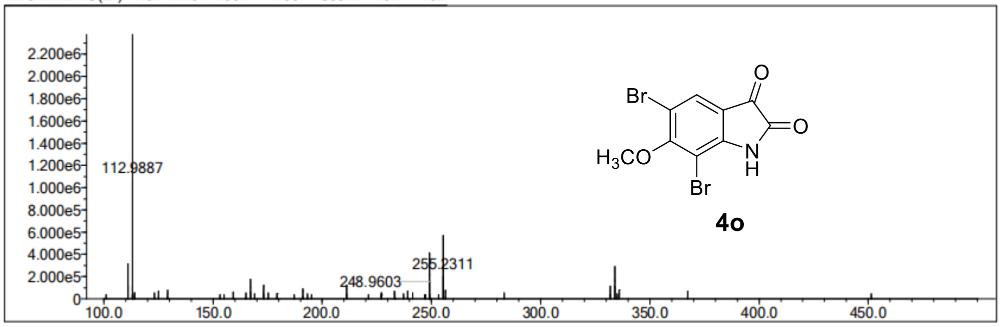


Measured region for 331.8547 m/z

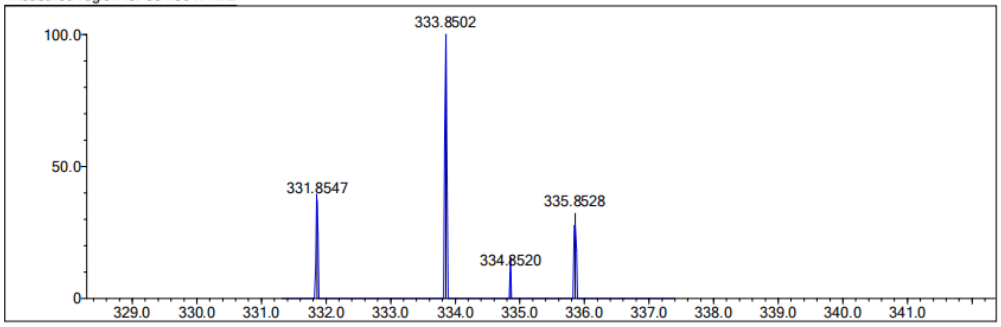


C9 H5 N O3 Br2 [M-H]- : Predicted region for 331.8563 m/z

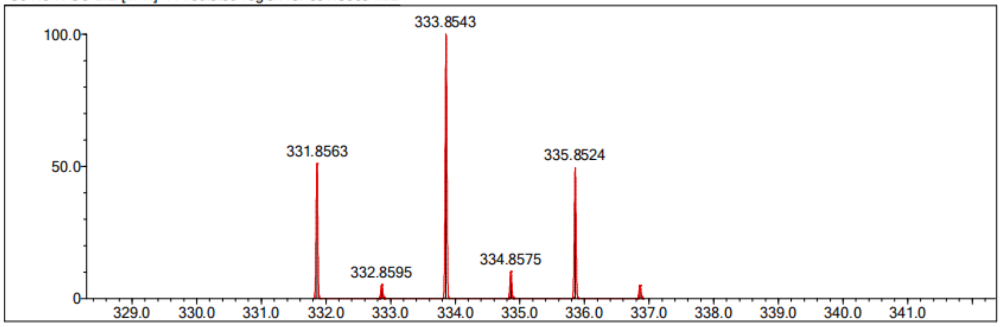


| Rank | Score | Formula (M) | Ion | Meas. m/z | Pred. m/z | Df. (mDa) | Df. (ppm) | Iso | DBE |
|---|---|---|---|---|---|---|---|---|---|
| 1 | 36.64 | C9 H5 N O3 Br2 | [M-H]- | 331.8547 | 331.8563 | -1.6 | -4.82 | 40.51 | 7.0 |
